# Long-term psychological effects of COVID-19-related quarantine: an observational study of three cohorts in Norway and Iceland

**DOI:** 10.1186/s12916-025-04349-8

**Published:** 2025-09-25

**Authors:** Li Lu, Yue Wang, Omid V. Ebrahimi, Qing Shen, Anna Bára Unnarsdóttir, Arna Hauksdóttir, Asle Hoffart, Edda Bjork Thordardottir, Ingibjörg Magnúsdóttir, Jóhanna Jakobsdóttir, Lill Trogstad, Thor Aspelund, Fang Fang, Ragnhild E. Brandlistuen, Sverre Urnes Johnson, Unnur Anna Valdimarsdóttir, Ole A. Andreassen, Helga Ask

**Affiliations:** 1 Centre for Precision Psychiatry, Institute of Clinical Medicine, University of Oslo, Oslo, Norway; 2 PsychGen Centre for Genetic Epidemiology and Mental Health, Norwegian Institute of Public Health, Oslo, Norway; 3 Centre of Public Health Sciences, Faculty of Medicine, School of Health Sciences, University of Iceland, Reykjavik, Iceland; 4 Department of Experimental Psychology, University of Oxford, Oxford, UK; 5 Modum Bad Psychiatric Hospital and Research Center, Vikersund, Norway; 6 Unit of Integrative Epidemiology, Institute of Environmental Medicine, Karolinska Institutet, Stockholm, Sweden; 7 Clinical Research Center for Mental Disorders, Shanghai Pudong New Area Mental Health Center, Tongji University School of Medicine, Shanghai, China; 8 Department of Psychology, University of Oslo, Oslo, Norway; 9 Division of Infection Control, Norwegian Institute of Public Health, Oslo, Norway; 10 The Norwegian Mother, Father and Child Cohort Study, Norwegian Institute of Public Health, Oslo, Norway; 11 Department of Epidemiology, Harvard TH Chan School of Public Health, Boston, MA USA; 12 Centre for Precision Psychiatry, Division of Mental Health and Addiction, Oslo University Hospital, Oslo, Norway; 13 Department of Mental Disorders, Norwegian Institute of Public Health, Oslo, Norway

**Keywords:** Depression, Anxiety, Quarantine, COVID-19, Longitudinal study, Multicohort study

## Abstract

**Background:**

Longitudinal assessments of psychological effects related to length and recency of quarantine experience in general populations are of importance. We aim to investigate if recency and duration of quarantine exposures during the COVID-19 pandemic were associated with mental health, across subgroups.

**Methods:**

We included three prospective cohorts from Iceland and Norway with data on quarantine and symptoms of depression and anxiety from March 2020 to March 2022. We calculated prevalence ratios (PR) of probable depression and anxiety in relation to quarantine exposure, and performed longitudinal analyses in a subpopulation with repeated assessments to test the potential change in mental health burden due to quarantine over time while controlling for current quarantine status and other covariates.

**Results:**

In total, 105,344 and 94,435 individuals were included in the analysis of probable depression and anxiety, respectively, with 18.2% and 40.0% reporting quarantine exposure before the most recent assessment of corresponding mental health symptoms. Overall, quarantine exposure was associated with probable depression (PR 1.19 [95% CI: 0.99–1.42]) and anxiety (PR 1.21 [1.08–1.36]). Compared to individuals without quarantine, being exposed to quarantine for 0–2, 2–4, or > 4 weeks was associated with incrementally higher prevalence of probable depression (PR 1.15 [0.93–1.43]; 1.34 [1.06–1.68]; 1.72 [1.35–2.18], respectively) and probable anxiety (PR 1.12 [1.01–1.23]; 1.28 [1.14–1.45]; 1.76 [1.56–1.98]) in a step-wise manner; those who were quarantined within the last 2 weeks, last 2–4 weeks, or earlier showed a higher prevalence of probable depression in a dose–response manner (PR 1.62 [1.32–1.99], 1.32 [1.07–1.63], and 1.23 [1.06–1.43], respectively). The prevalence of probable anxiety did not appear to differ by the recency of quarantine. The longitudinal analyses (mean follow-up: 20.5 months) confirmed significantly higher prevalence of probable depression but only among those who were quarantined for > 4 weeks (PR 1.61 [1.30–2.00]), and of probable anxiety among those quarantined 2–4 weeks (PR 1.29 [1.14–1.45]) and > 4 weeks (PR 1.56 [1.34–1.82]).

**Conclusions:**

This study underscores the importance of monitoring mental well-being of populations recently quarantined, particularly those quarantined for prolonged periods. Greater emphasis should be placed on the detrimental psychological effects in the risk-cost–benefit analysis of quarantine as a mitigation strategy in future pandemics.

**Supplementary Information:**

The online version contains supplementary material available at 10.1186/s12916-025-04349-8.

## Background

The COVID-19 pandemic has, to date, adversely affected millions of lives globally, impacting both short- and long-term health and well-being following infection with the SARS-CoV-2 virus [1, 2]. Quarantine is a frequent social mitigating strategy used to control the rapid spread of contagious diseases during epidemics or pandemics, implemented through governmental recommendations restricting outdoor activities, travel, enforcing home confinement, and lockdowns [3]. While quarantine is an efficient way to reduce spread of infection [3, 4], it can have detrimental mental health effects such as accentuated stress, anxiety, and depression [5]. During the COVID-19 pandemic, countries adopted different physical distancing strategies to balance health protection [6]. For example, during the first year of the pandemic, Norway imposed population movement restrictions that included enforced home office where possible, home schooling, closures of targeted businesses, restaurants closures, and the closing of international borders to non-residents [7]. Iceland imposed similar yet somewhat less stringent gathering restrictions compared to Norway, but rather focused on large-scale testing and contact tracing with physical distancing and mandatory quarantine requirements to limit virus spread within the community [8]. These various mitigation methods may translate into different emotional reactions and mental health burdens [9]. Although evidence has accumulated suggesting an increased mental health burden related to COVID-19 infection [2], vaccination hesitancy [10], and residential mobility restriction [11], it remains unclear how the quarantine experience, such as duration (i.e., overall length exposed to quarantine) and recency of quarantine exposure, may impact the mental health of affected individuals.

Existing studies have mainly reported negative psychological health related to quarantine at one specific time point, such as post-traumatic stress symptoms, confusion and anger, as well as symptoms of anxiety and depression [3, 12–16]. Associations have been reported among selected groups of people (e.g., infected individuals, young people or university students) [16–18], using varying measures of quarantine experiences (Bonati et al., 2022) and mainly cross-sectional designs [19, 20], leaving the findings vulnerable to contextual confounders and differences in measurements over time. Studies conducted in the early stages of the pandemic suggested a relatively small impact of quarantine on mental health [21]. Thus, investigating impact of quarantine surpassing the early stages of the pandemic is warranted due to the possible hazards of experiencing these invasive measures over longer spans of time [3, 7]. Accordingly, while longer quarantine duration could serve as a potential risk factor for mental health outcomes [3], longitudinal studies mapping the mental health effects of quarantine in the general population during the COVID-19 pandemic are scarce [22–24], which should be explored by duration of quarantine especially with longitudinal design. It further remains unclear whether individuals experiencing mental health burdens due to COVID-19 quarantine recover after the elimination of these invasive protocols, since those who have recently undergone quarantine may still be dealing with unresolved stressors, biological stress responses, and disrupted social connections. Therefore, exploring quarantine from a comprehensive perspective such as including duration and recency can help to better capture the temporal dynamics of psychological distress, cumulative stress, and design strategies to mitigate its adverse effects while promoting resilience and well-being.

Leveraging data from three large longitudinal cohort studies in Iceland and Norway (with approximately 100,000 participants), as part of the COVIDMENT Consortium [25], we aimed to investigate how the recency and duration of quarantine exposures during the COVID-19 pandemic were associated with mental health, specifically symptoms of depression and anxiety, across gender and age groups. We further utilized repeated measures of the mental health outcomes to assess changes in the prevalence of depressive and anxiety symptoms before and after being quarantined in a subset of the cohorts. By integrating four constructed variables presenting quarantine exposures, we here attempt to provide comprehensive evidence on safe vs. non safe duration of quarantine, as well as psychological recovery time after quarantine in order to aid the organization of effective pandemic mitigation strategies with minimal negative psychological effects. This approach could benefit policy-making and implementation in future pandemic governance.

## Methods

### Study design and participants

This study included three cohorts from the COVIDMENT Consortium with data available on recency and duration of quarantine, and an extensive longitudinal follow-up of depressive and anxiety symptoms from March 2020 to March 2022 (up to 24 months), including the Icelandic COVID-19 National Resilience Cohort (C-19 Resilience, data collection from April 2020 through July 2021), the Norwegian COVID-19 Mental Health and Adherence Study (MAP-19, March 2020 to March 2022), and the Norwegian Mother, Father, and Child Cohort Study (MoBa, March 2020 through March 2021) [25].

C-19 Resilience was established in April 2020 and eligible for all adult Icelandic-speaking residents with specific outreach to participants of ongoing cohorts for participation. The participants signed an electronic informed consent by unique Bank ID (electronic ID confirmation system), and then answered an extensive web-based baseline questionnaire (April through July 2020) with two follow-up questionnaires in December 2020–March 2021 and May 2021–July 2021 [25]. Recruitment of the MAP-19 cohort was conducted through targeting a random selection of all Norwegian adults available on Facebook (85% of all Norwegian adults) using a Facebook algorithm, who subsequently made up 70% of the participants of MAP-19, from the baseline survey between March 2020 and April 2020 to last assessment wave in March 2022. To reach the remaining 15% of the Norwegian population not on Facebook, the survey was broadcasted systematically across national, regional, and local media platforms (e.g., TV, radio, and newspapers) [26]. In MoBa, web-based questionnaires were sent to all adult participants in the existing cohort biweekly to collect COVID-19-related information from March 2020 onward [27]. In all three cohorts, data of the studied mental health outcomes at the first and most recent measurement among the COVID-19 surveys was used for the current study.

### Data collection and assessment

#### Exposure variables

Information on quarantine exposures were originally self-reported in all cohorts.

- In the C-19 Resilience cohort, participants were asked in all data collections: “Have you been in quarantine due to COVID-19?”
- In MoBa, participants were asked biweekly (from April 2020 to July 2021): “Have you been in quarantine/isolation during the last 14 days?”
- In MAP-19, it was asked: “How many times have you been in quarantine due to COVID-19?” Quarantine duration was calculated assuming the participants were quarantined for 10 days each time (according to the guidelines).

Based on the collected information from the three cohorts, we constructed the following four exposure variables representing quarantine experience: (1) ever reporting exposure to quarantine experience before the most recent assessment of the mental health outcomes (*quarantine experience ever*), coded as 1 (yes) or 0 (no); (2) *duration of quarantine*, reflecting how many weeks the participant had been quarantined in total before the most recent assessment of the mental health outcomes, categorizing as no quarantine, 0–2 weeks (≤ 14 days), 2–4 weeks (15–28 days), or > 4 weeks (> 28 days); (3) *time since the end of the most recent quarantine (recency of quarantine)*, indicating time between the most recent quarantine and the time of the most recent assessment of the mental health outcomes, categorizing as no quarantine, 0–2 weeks, 2–4 weeks, or > 4 weeks; and (4) *total duration of quarantine between the first and the most recent measurements of the mental health outcomes*, grouping as no quarantine, 0–2 weeks, 2–4 weeks, or > 4 weeks. The first three exposure variables were treated as independent variables in cross-sectional analyses, and the fourth was included in the longitudinal analysis. Notably, participants with quarantine exposure before the first measurement of mental health outcomes were excluded from the longitudinal analysis. The dates, assessments of which quarantine information was collected in each cohort, and constructed exposure variables are shown in Table S1 in Additional file 1. The quarantine policies over time in Norway and Iceland are shown in Table S2 in Additional file 1, specifically, as indicated by previous study [28], despite the similarities in adopted policy measures, quarantine policies such as requirements for quarantine and length of quarantine were changed at different timepoints between countries.

#### Outcome variables

We used the validated 7-item Generalized Anxiety Disorder Scale (GAD-7) to assess level of anxiety symptoms experienced during the last 2 weeks [29, 30]. Each item has response options scored from 0 (“not at all”) to 3 (“nearly every day”). The sum score of the 7 items ranges from 0 to 21, with higher scores indicating more symptoms. Consistent with previous studies [29, 31], we defined those with a sum score of 10 or greater as having moderate or severe anxiety symptoms, i.e., probable anxiety. Depressive symptoms during the past 2 weeks were measured using the validated Patient Health Questionnaire-9 (PHQ-9) [32]. Participants indicated how often they had been bothered by each symptom using a four-point Likert scale ranging from 0 (not at all) to 3 (nearly every day), summing up to an overall score of 0 to 27. A sum score of 10 or greater maximizing combined sensitivity and specificity were considered to have moderate or severe depressive symptoms, i.e., probable depression [32, 33].

Data on mental health outcomes collected at the first and the most recent measurement was used in this study. The dates for exposure and outcome information is provided in Table S1 in Additional file 1 for each cohort.

#### Covariates

We included the following covariates when available in each cohort: gender (male, female, other), age at enrollment (i.e., baseline; < 35 years, 35–44, 45–54, 55–64, 65 years or older; 35–44, 45–54, and 55–64 were merged into 35–64 years in the subgroup analyses), highest level of attained education (no formal education; compulsory; upper secondary, vocational, or other; bachelor’s; diploma university degree; Master’s or Ph.D.), body mass index (BMI, categorized as < 25 kg/m^2^ [normal or low weight], 25–30 kg/m^2^ [overweight], > 30 kg/m^2^ [obese]), current smoking (no or yes), history of psychiatric disorder (no or yes), chronic medical conditions (no, one, two or more comorbidities), COVID-19 diagnosis (no or yes; self-reported SARS-CoV-2 infection from a positive RT-PCR test or positive antigenic self-test, or ever been diagnosed with COVID-19 until the most recent measurement of mental health), and living condition (living alone or living with other people; not available in C-19 Resilience) before the most recent measurement of mental health outcomes. Current quarantine status (yes/no) and current living condition (not available in C-19 Resilience) reported at the most recent measurement of mental health outcomes were also included as covariates, aiding in the promotion of inferential validity of the relationship between previous quarantine exposure and current mental health status.

Number of children in need of care (0, 1, or 2 and more), binge drinking (yes/no; by asking “in the past 2 months, how many alcoholic beverages did you typically have when you drank?”, it was defined as binge drinking if females drink 4 or more drinks typically and males 5 or more drinks typically), personal monthly income (1 = equal or less than 300 thousand, 2 = 301–500 thousand, 3 = 501–700 thousand, 4 = 701–1000 thousand, 5 = more than a million; by asking “in what range do you estimate your total monthly income has been on average in the past 12 months [in ISK]?”), and employment status (yes/no; full time employee) at baseline were available in the C-19 Resilience cohort and were additionally adjusted for in the sensitivity analysis. Covariate availability in each cohort is summarized in Table S3 in Additional file 1.

### Statistical analyses

In all analyses, we used robust Poisson regression with quasi-likelihood models [34] to study the binary outcomes of probable anxiety and depression. The models provide prevalence ratios (PRs) with 95% confidence intervals (CIs), comparing the prevalence of probable anxiety and depression between groups with different quarantine exposure. A sandwich estimator with exchangeable working correlation structure was applied in the models to control for intra-individual correlation when repeated measures were used [35]. We calculated PRs in crude models and adjusted models by controlling for the above listed covariates.

We first performed all analyses using a standardized protocol in the individual cohorts, and then meta-analyzed the cohort-specific results with a random-effects model using the “*metafor*” package [36] in R.* I*^2^ statistic was used to examine the heterogeneity of the cohort-specific findings [37]. We additionally conducted trend analyses with the “rma” function within “*metafor*” package to test whether the trend for each outcome among the three cohort was statistically significant.

#### Cross-sectional analyses

First, we ran a model including quarantine experience (yes/no) as exposure and probable anxiety and depression (no or yes) as outcomes. We then performed stratified analyses to explore possible effect modifiers (source of heterogeneity) of the studied associations, including gender, age (< 35, 35–64, 65 years or more), history of psychiatric disorder, and previous COVID-19 diagnosis. Second, we conducted analyses to examine whether the studied associations would differ by (1) *duration* and (2) *recency of quarantine*.

#### Longitudinal analyses

In a subpopulation of participants without quarantine experience before the first measurement of mental health outcomes and with repeated measures of anxiety (all three cohorts) and depressive (C-19 Resilience and MAP-19) symptoms during the study, we conducted a longitudinal analysis to test the potential change in mental health burden over time. A pairwise analysis was performed to assess the PRs of probable anxiety and depression in relation to different durations of quarantine between the two measurements of mental health outcomes, compared with participants without quarantine experience. The analyses were adjusted for the same covariates as above.

In all analyses, missing values were imputed through multiple imputation by chained equations using the “*mice*” package in R [38]; the percentage of missingness across different variables ranged from 0% to 17.5%, with the only exception being the BMI at 26.8% among MoBa participants with data on depressive symptoms (see Table 1 for details).

**Table 1:** Descriptives of the included participants of the three cohorts

	**Iceland**	**Norway**		
	**C-19 Resilience**	**MAP-19**	**MoBa**^c^	
	*n* (%)	*n* (%)	**Anxiety symptoms**	**Depressive symptoms**
			*n* (%)	*n* (%)
**Total**	10,431	2963	81,041	91,950
**Gender**				
Female	7118 (68.2%)	2285 (77.1%)	49,736 (61.4%)	55,411 (60.3%)
Male	3204 (30.7%)	672 (22.7%)	31,305 (38.6%)	36,539 (39.7%)
Other	14 (0.1%)	6 (0.2%)	0 (0%)	0 (0%)
Missing	95 (0.9%)	0 (0%)	0 (0%)	0 (0%)
**Age**				
Mean. years (SD)	55.8 (13.5)	38.1 (13.9)	47.2 (5.21)	47.0 (5.22)
< 35 years	871 (8.4%)	1467 (49.5%)	301 (0.4%)	429 (0.5%)
35–44 years	1213 (11.6%)	624 (21.1%)	23,594 (29.1%)	27,532 (29.9%)
45–54 years	2346 (22.5%)	433 (14.6%)	48,322 (59.6%)	54,205 (59.0%)
55–64 years	2938 (28.2%)	276 (9.3%)	5807 (7.2%)	6350 (6.9%)
65 years or more	3063 (29.4%)	163 (5.5%)	282 (0.3%)	272 (0.3%)
Missing	0 (0%)	0 (0%)	2735 (3.4%)	3162 (3.4%)
**Education**				
No formal education	0 (0%)	4 (0.1%)	0 (0%)	0 (0%)
Compulsory	1360 (13.0%)	136 (4.6%)	1403 (1.7%)	1673 (1.8%)
Upper secondary, vocational, or other	3165 (30.3%)	1061 (35.8%)	22,165 (27.4%)	25,849 (28.1%)
Bachelor’s/diploma university degree	3331 (31.9%)	1762 (59.5%)^a^	28,925 (35.7%)	32,248 (35.1%)
Master’s or Ph.D	2439 (23.4%)	-	23,954 (29.6%)	26,816 (29.2%)
Missing	136 (1.3%)	0 (0%)	4594 (5.7%)	5364 (5.8%)
**BMI (kg/m**^**2**^**)**				
< 25, normal or low weight	2924 (28.0%)	1317 (44.4%)	29,887 (36.9%)	30,066 (32.7%)
25–30, overweight	3909 (37.5%)	984 (33.2%)	25,290 (31.2%)	25,530 (27.8%)
> 30, obese	3266 (31.3%)	662 (22.3%)	11,712 (14.5%)	11,697 (12.7%)
Missing	332 (3.2%)	0 (0%)	14,152 (17.5%)	24,657 (26.8%)
**Current smoking status**				
No	8777 (84.1%)	2752 (92.9%)	68,089 (84.0%)	80,338 (87.4%)
Yes	1468 (14.1%)	211 (7.1%)	6815 (8.4%)	8417 (9.2%)
Missing	186 (1.8%)	0 (0%)	6137 (7.6%)	3195 (3.5%)
**History of psychiatric disorder**				
No	7280 (69.8%)	2280 (76.9%)	65,418 (80.7%)	74,135 (80.6%)
Yes	2908 (27.9%)	683 (23.1%)	12,819 (15.8%)	14,563 (15.8%)
Missing	243 (2.3%)	0 (0%)	2804 (3.5%)	3252 (3.5%)
**Pre-existing comorbidity**				
No	5720 (54.8%)	1969 (66.5%)	63,190 (78.0%)	73,323 (79.7%)
One comorbidity^b^	3143 (30.1%)	994 (33.5%)	11,668 (14.4%)	13,543 (14.7%)
Two or more comorbidities	1416 (13.6%)	0 (0%)	1667 (2.1%)	1921 (2.1%)
Missing	152 (1.5%)	0 (0%)	4516 (5.6%)	3163 (3.4%)
**COVID-19 infection**				
Not infected	9870 (94.6%)	1511 (51.0%)	80,034 (98.8%)	91,743 (99.8%)
Infected	549 (5.3%)	1452 (49.0%)	1007 (1.2%)	207 (0.2%)
Missing	12 (0.1%)	0 (0%)	0 (0%)	0 (0%)

^a^Any university degree

^b^For MAP-19, data of one comorbidity or more was available

^c^The first wave of anxiety symptoms was assessed at the same time periods as that for depressive symptoms for both C-19 Resilience and MAP-19, i.e., Apr 2020–July 2020 for C-19 Resilience and Mar 31–Apr 7, 2020 for MAP-19. While for MoBa, the first wave assessment of anxiety symptoms was between May 12 and May 27, 2020, and of depressive symptoms was between June 10 and June 24, 2020, which were presented separately

#### Sensitivity analyses

In sensitivity analyses, we additionally adjusted the cross-sectional analyses for the number of children in care and binge drinking in step 1, and personal monthly income and employment status in step 2 that were available in the C-19 Resilience cohort, to assess potential residual confounding, since the four individual-level factors could interact with the social environment (such as quarantine) to collectively influence mental health. And we performed the cross-sectional analyses among individuals with complete information on all covariates in the MoBa cohort, to assess the impact of multiple imputation. All data analyses were performed in R version 4.1.2. We reported this study according to the Strengthening the Reporting of Observational studies in Epidemiology (STROBE) checklist [39].

### Role of the funding source

The funders had no role in study design, data collection, data analysis, interpretation, or writing of the report.

## Results

### Sample descriptions

In total, 94,435 (female: 62.6%; mean age: 47.9 ± 7.7 years) and 105,344 (female: 61.5%; 47.6 ± 7.5 years) participants were included in the analysis for probable anxiety and probable depression, respectively. Table 1 shows the characteristics of the study participants from each cohort (C-19 Resilience: 10,431; MAP-19: 2963; MoBa: 81,041 and 91,950 with data on anxiety and depressive symptoms, respectively). The characteristics varied between the cohorts, with a lower mean age at enrollment in MAP-19 (38.1 years) compared to other cohorts (e.g., 55.8 years in C-19 Resilience). All cohorts were over-represented by female participants, with a proportion ranging from 60.3% (MoBa) to 77.1% (MAP-19). Participants in C-19 Resilience cohort had a higher mean age and higher prevalence of overweight and obesity, current smoking, history of psychiatric disorder, and pre-exiting comorbidities, than other cohorts (Table 1).

The prevalence of probable depression and probable anxiety at the baseline were 15.5% (95% CI: 14.8%–16.2%) and 10.4% (95% CI: 9.8%–11.0%) for C-19 Resilience, 29.5% (95% CI: 27.8%–31.3%) and 17.6% (95% CI: 16.2%–19.0%) for MAP-19, and 4.9% (95% CI: 4.7%–5.0%) and 3.6% (95% CI: 3.5%–3.7%) for MoBa (see Table S4 in Additional file 1 for the prevalence at the end point). The characteristics of study participants by the length of quarantine before the most recent assessment of mental health outcomes are shown in Table S5 in Additional file 1 for different cohorts. In total, before the most recent assessment of anxiety symptoms (C-19 Resilience: May–July 2021; MAP-19: March 2022; MoBa: March 2021), 40.0% of the participants reported quarantine exposure (22.8%, 10.2%, and 6.7% for individuals with 0–2 weeks, 2–4 weeks, and more than 4 weeks quarantine, respectively), and before the most recent assessment of depressive symptoms (C-19 Resilience: May–July 2021; MAP-19: March 2022; MoBa: June 2020), the corresponding exposure prevalence was 18.2% (13.7%, 3.1%, and 1.4% for 0–2 weeks, 2–4 weeks, and > 4 weeks quarantine, respectively).

### Cross-sectional analyses

**Fig. 1:**
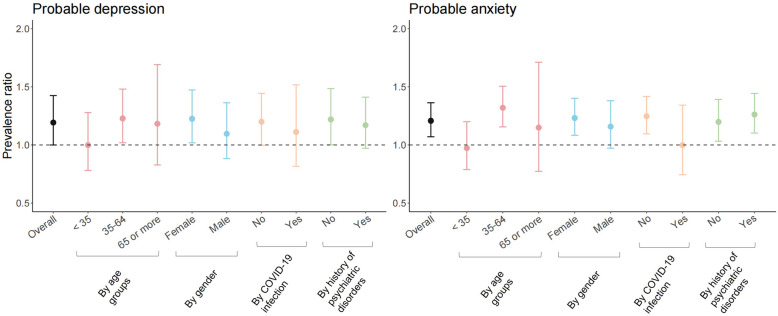
Prevalence ratio (PR) and 95% confidence interval (CI) of probable depression (left) and possible anxiety of participants with *quarantine experience ever* compared with those without quarantine. Each line represents result from one model *indicating the overall PR and PRs stratified by age group, sex, COVID-19 infection, and history of psychiatric disorder*. Adjustment variables: age group, gender, education, BMI, current smoking, history of psychiatric disorder, chronic medical conditions, COVID-19 diagnosis, living condition before the latest measurement of mental health indicators, current quarantine and living condition. Age-stratified models: adjusted for adjustment variables—age group. Gender-stratified models: adjusted for adjustment variables—gender. COVID-19 infection-stratified models: adjusted for adjustment variables—COVID-19 diagnosis. History of psychiatric disorder-stratified models: adjusted for adjustment variables—history of psychiatric disorder

The meta-analysis showed that compared to individuals without quarantine, individuals with quarantine exposure exhibited a higher prevalence of probable anxiety (pooled adjusted overall PR: 1.21 [95% CI: 1.08–1.36]), while the likelihood of a higher exposure prevalence of probable depression was marginally significant (pooled adjusted overall PR: 1.19 [0.999–1.42]; Fig. 1; please see results of crude model in Table S6 in Additional file 1). Subgroup analyses indicated that these associations were pronounced among participants aged 35–64 years, female participant, and participants without COVID-19 infection. The associations were generally more pronounced in MoBa and less clear in MAP-19 (Figs. S1 and S2 in Additional file 1 for probable depression and anxiety in each cohort, respectively).

#### Duration of quarantine

**Fig. 2:**
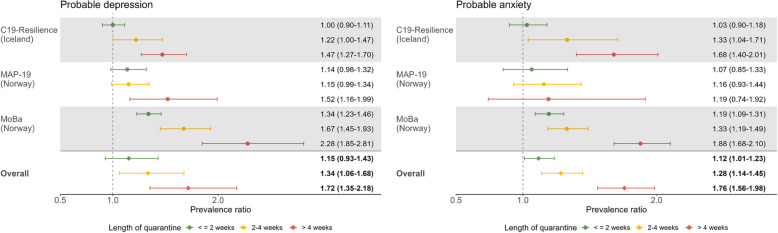
Prevalence ratio (PR) and 95% confidence interval (CI) of probable depression (left) and probable anxiety (right) among individuals with different *duration of quarantine* compared with those *without quarantine*. Adjusted for age group, gender, education, BMI, current smoking, history of psychiatric disorder, chronic medical conditions, COVID-19 diagnosis, living condition before the latest measurement of mental health indicators, current quarantine and living condition

Compared with participants with no quarantine exposure, longer duration in quarantine was associated with a higher prevalence of both probable anxiety and depression in a dose–response manner (Fig. 2). Please find results of crude model in Table S6 in Additional file 1. Being quarantined for 0–2 weeks, 2–4 weeks, or > 4 weeks was associated with a higher prevalence of probable depression (*n* = 104,793, adjusted overall PR: 1.15 [0.93–1.43]; 1.34 [1.06–1.68]; 1.72 [1.35–2.18], respectively; *I*^2^ = 86.5%, *p* < 0.001), as well as probable anxiety (*n* = 93,884, adjusted overall PR: 1.12 [1.01–1.23]; 1.28 [1.14–1.45]; 1.76 [1.56–1.98], respectively; *I*^2^ = 29.6%, *p* = 0.18). The trend analyses showed statistical significance (in Table S7 in Additional file 1; probable depression: *Z*_*trend*_ = 0.294, *p*_*trend*_ < 0.001; probable anxiety: *Z*_*trend*_ = 0.318, *p*_*trend*_ < 0.001). Similar patterns of associations were observed across the three cohorts; however, in MAP-19, the only statistically significant association was observed in the relationship between being quarantined for more than 4 weeks and probable depression.

#### Recency of quarantine

**Fig. 3:**
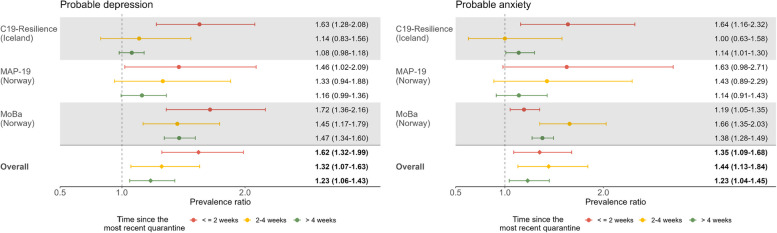
Prevalence ratio (PR) and 95% confidence interval (CI) of probable depression (left) and probable anxiety (right) by *time since the most recent quarantine* (compared with those without quarantine). Adjusted for age group, gender, education, BMI, current smoking, history of psychiatric disorder, chronic medical conditions, COVID-19 diagnosis, living condition before the latest measurement of mental health indicators, current quarantine and living condition

The results further indicated that time passed since the most recent quarantine exposure was associated with a decreasing prevalence of probable depression, indicating that more recent exposure to quarantine was associated with a higher prevalence (Fig. 3). Results of crude models are shown in Table S6 in Additional file 1. Compared with individuals without quarantine experience, those who were quarantined within the last 2 weeks, last 2–4 weeks, or more than 4 weeks earlier showed a higher prevalence of probable depression in a dose–response manner (*n* = 82,888; adjusted PR: 1.62 [95% CI: 1.32–1.99], 1.32 [95% CI: 1.07–1.63], and 1.23 [95% CI: 1.06–1.43], respectively; *I*^2^ = 66.5%, *p* < 0.001; *Z*_*trend*_ = − 0.190, *p*_*trend*_ = 0.001). The prevalence of probable anxiety did not appear to differ significantly by time since the most recent quarantine exposure (*n* = 94,147; 1.35 [95% CI: 1.09–1.68], 1.44 [95% CI: 1.13–1.84], and 1.23 [95% CI: 1.04–1.45], respectively; *I*^2^ = 62.7%, *p* = 0.01; *Z*_*trend*_ = − 0.081, *p*_*trend*_ = 0.513).

Sensitivity analyses additionally adjusting for number of children in care and binge drinking, and personal monthly income and employment status in the C-19 Resilience cohort showed similar estimates trends (with point estimates have changed slightly) (Table S8 in Additional file 1). The estimates were also similar in the MoBa cohort after excluding participants with missing values (in Table S9 in Additional file 1).

### Longitudinal analyses

**Fig. 4:**
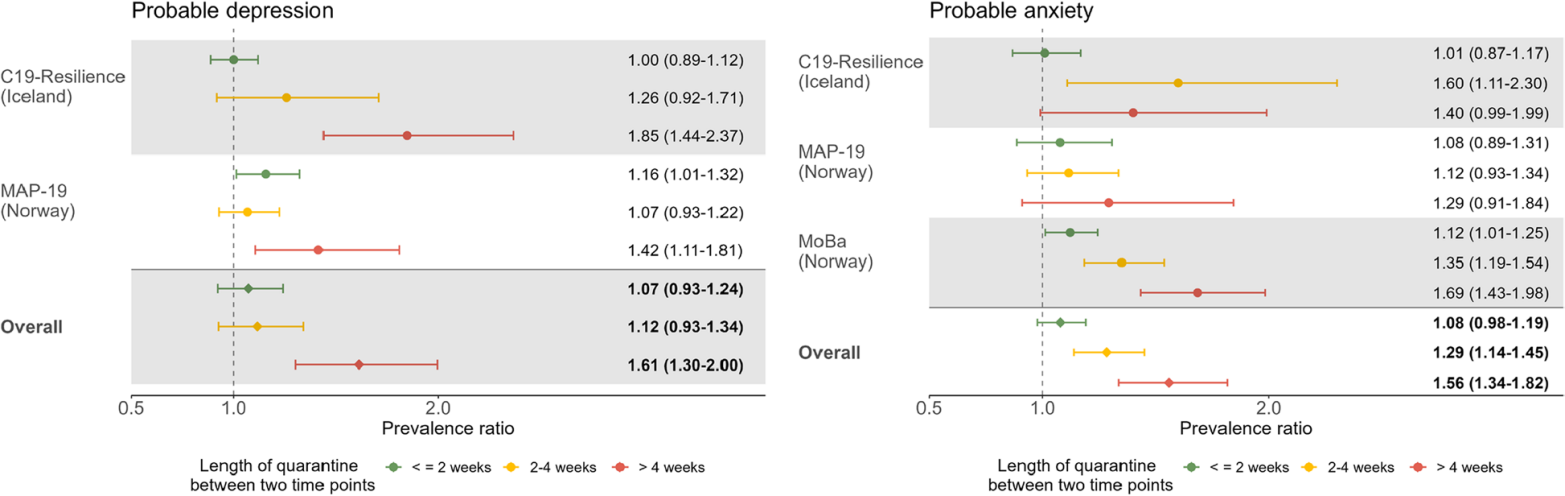
Longitudinal change in the prevalence of probable depression (left) and probable anxiety (right) by the total duration of quarantine between two measurements. Note: *Two measurements*, the first (not included) and the most recent (not included) mental health measurement. Adjusted for age group, gender, education, BMI, current smoking, history of psychiatric disorder, chronic medical conditions, COVID-19 diagnosis, living condition before the latest measurement of mental health indicators, current quarantine and living condition

The pairwise analysis of participants with repeated measures of depressive (available for C-19 Resilience and MAP-19; *N* = 8051; time interval = 10.2–24.9 months, mean time interval = 20.5 months) and anxiety symptoms (available for all cohorts, *N* = 64,311; time interval = 9.7–24.9 months, mean time interval = 16.9 months) largely confirmed the results of the cross-sectional analysis (Fig. 4). Compared with participants who had no quarantine between the two measurements, a statistically significantly higher pooled prevalence of probable depression was noted for those who were quarantined for more than 4 weeks (adjusted PR: 1.61, 95% CI: 1.30–2.00; *Z*_*trend*_ = 0.290, *p*_*trend*_ = 0.013). Additionally, individuals quarantined for more than 2 weeks presented a statistically significantly higher prevalence of probable anxiety (2–4 weeks, PR: 1.29 [95% CI: 1.14–1.45]; > 4 weeks, 1.56 [95% CI: 1.34–1.82]; *I*^2^ = 20.6%, *p* = 0.26; *Z*_*trend*_ = 0.258, *p*_*trend*_ < 0.001) (Fig. 4; Table S5 in Additional file 1 displays the results for the crude models).

## Discussion

Leveraging data from three large longitudinal cohorts from Iceland and Norway, we found an elevated prevalence of probable depression and anxiety among individuals exposed to quarantine during the COVID-19 pandemic. The association was dose-dependent with duration of quarantine, with the most pronounced prevalence increase noted among individuals quarantined for 4 weeks or more, especially confirmed by longitudinal analysis with up to 24 months follow-up. This association between psychological health and quarantine was greater among females, middle-aged individuals, and those without a COVID-19 infection, and was both noted among individuals with and without a previous diagnosis of mental disorder. The increase in the prevalence of probable depression and anxiety remained significant over 4 weeks after being released from quarantine. Our findings shed further light on the psychological burden associated with quarantine which should be considered in future planning and organization of containment measures. Tailored and targeted prevention and intervention strategies for specifically vulnerable subgroups should also be considered.

While quarantine exposure has been associated with adverse mental health symptoms during the COVID-19 pandemic [7, 20, 40, 41], less was known about how the duration of quarantine affects symptoms of depression and anxiety, and whether these symptoms attenuate over time after quarantine exposure. In our study, we found that the length of quarantine exposure was significantly associated with increased prevalence of probable depression and anxiety in a dose-dependent manner, with longer periods in quarantine (i.e., 4 weeks or more) yielding the most pronounced prevalence increase. In line with our results, findings by Hawryluck et al. [42] from the SARS pandemic indicated that individuals quarantined for more than 10 days experienced higher levels of depressive symptoms compared to those quarantined for less than 10 days. Although the prevalence among individuals with different durations of quarantine was elevated compared to those without quarantine in three cohorts, the magnitude of this association was higher among participants from the MoBa cohort, than MAP-19 and C-19 Resilience cohorts. This difference may be attributed to that MoBa specifically recruited parents who may have experienced greater family burden and responsibilities when quarantined. The increase in psychological distress with extended duration of quarantine may have several explanations [3, 43]. A recent study hypothesized that immobility is one possible pathway through which quarantine may be related to depression and anxiety [13]. Longer periods of immobility due to longer quarantine periods may lead to reduced physical activity, which is a known risk factor of depression and anxious symptomatology [44, 45]; and in another points that it could lead to more time to ruminate, a key process in the development of depression [46]. Additionally, longer periods of quarantine may be tied with increased social disconnection and loneliness, both of which have been associated with mental health adversities including increasing depressive symptoms during the COVID-19 pandemic [47, 48].

Recent quarantine exposure (within the last 2 weeks) was associated with the greatest probable depression and anxiety burden, with some indications of change over time, although these symptoms remained statistically elevated after even 4 weeks post-quarantine exposure. The mental health symptoms were assessed over the past 2 weeks, thus the association with quarantine within the last 2 weeks may indicate the time they were in quarantine, a period of possible infection, adverse physical and mental health. As time passes after quarantine exposure, individuals gradually return to their normal routines, including exercise and physical activity, which, as previously mentioned, were associated with reductions in depressive symptoms [44, 45]. However, a significantly decreased trend was not observed in anxiety symptoms, especially among MoBa participants, who were all parents. This may be due to the ongoing burdens of normal routines and childcare responsibilities, which should be further investigated once more data becomes available.

We found that females may be more vulnerable for negative mental health impacts of quarantine compared to males. Previous studies have demonstrated gender differences in work-load at home during the pandemic such as the additional caregiver and household responsibilities that predominantly fall on women [49, 50]. Additionally, females may be more rely on social support during stressful periods, which may not be as readily available when in quarantine [51, 52]. Moreover, we further found the increase in prevalence of probable depression and anxiety after quarantine was less pronounced among the youngest and oldest age groups. In addition, quarantine exposure could result in changes in family dynamics, such as daily routine disruptions, financial strain, and reduced personal space, potentially leading to increased marital dissatisfaction and family conflicts [53, 54], as well as domestic violence [55, 56], all of which may contribute to psychological distress. This is particularly relevant for middle-aged participants from the MoBa cohort, who were all parents and may need different dimensions of support to maintain their physical and mental health during such times [53]. Indeed, domestic and intimate partner violence increased significantly during the pandemic, potentially leaving individuals in quarantine in vulnerable situations [57, 58]. Potential mechanisms (e.g., social support, psychological resilience, coping strategies) should be further investigated. Interestingly, our results indicate that quarantine exposure was more strongly associated with probable anxiety among individuals without a COVID-19 infection. A possible explanation for this result is these individuals may have experienced more fear, uncertainty and possible frustration related to further infection, or feelings of senselessness on quarantine since they were not infected/ill, during the quarantine, compared to those who actually had been infected and quarantined with more explicit reasons [15, 59]. Notedly, insurance for healthcare and compensation for unemployment is mandatorily covered by all residents in Nordic countries, which ensures basic living conditions for temporary economic downturns. Taken together, our findings highlight the varying impact of quarantine on different subgroups of the general population, underscoring the need for tailored and targeted strategies to mitigate the negative psychological effects of quarantine.

### Strengths and limitations

The strengths of this study include the large sample size of participants reporting not only exposure to but also duration of quarantine, across three cohorts from two Nordic countries. In addition, the validated questionnaires used to quantify the severity of depressive and anxiety symptoms across cohorts enabled us to cross-validate findings across settings and in countries using different mitigation strategies, revealing the highlighting association of quarantine exposure across different contexts. Finally, we constructed different indicators for quarantine experience, i.e., the duration of quarantine (repeated measurements) and time since the most recent quarantine, which provided a multidimensional investigation of quarantine exposure using both a cross-sectional and longitudinal approach.

This study also has several limitations. First, the information on depressive and anxiety symptoms and quarantine exposure was self-reported, which could have introduced recall bias and social desirability bias, potentially leading to misestimations of the associations. However, we used measurement instruments that has been previously validated with high correspondence with registry-based outcomes, and the requirement for recall was low (at least for MoBa), as data collections happened every other week, which could help to moderate the magnitude of bias. Second, the depressive and anxiety symptoms were assessed at varying time periods across the three cohorts, coinciding with different pandemic waves (e.g., different virus variants and mitigation measures). These variations might influence both the prevalence of probable depression and anxiety as well as quarantine experience across cohorts. Third, while the Icelandic sample was selected from the general population, MoBa is restricted to parents giving birth to a child between 2000 and 2009, meaning that most of them (around 90%) were middle-aged (35–64 years) during the COVID-19 data collections, and the sample size for certain subgroups such as those aged 65 years or more is small. This might limit the generalizability of our findings to other age groups. Fourth, the cross-sectional analysis precludes inferences on temporality between quarantine exposure and mental health outcomes. Fifth, some of the remaining associations might be explained by other social-economic factors like income or employment status, or the location of quarantine, since those who quarantined at home might involve different stressors (e.g., family dynamics) compared to those quarantined in a hotel or hospital (e.g., lack of familiarity or medical environment), which should be considered in future research. Finally, the mandatory quarantine requirements varied by indication as well as in length and over time between Norway and Iceland, which may affect the results. However, the overall similar pattern observed between the Icelandic and Norwegian cohorts suggests minimal influence on our results.

## Conclusions

These data suggest that individuals exposed to a quarantine of 4 weeks or longer present higher prevalence of probable depression and anxiety. Although the prevalence of depression and anxiety decreases with time from the quarantine exposure, an elevated prevalence of these adverse symptoms was still noted more than 4 weeks past the discontinuation of the quarantine. These findings underscore the importance of monitoring the mental well-being of quarantined individuals and that negative psychological effects of extended quarantine should be considered in mitigation strategies of future pandemics and their risk-cost–benefit analyses.

## Supplementary Information

Additional file 1: Supplementary material.

## Data Availability

YW, TA and UAV had full access to the Icelandic data, HA had full access to the Norwegian MoBa data, and OVE and SUJ had full access to the Norwegian MAP-19 data, and take responsibility for the integrity and the accuracy of the data in the respective cohort. C-19 Resilience data cannot be made publicly available but interested researchers can get access to specific datasets by emailing the principal investigator (unnurav@hi.is) upon an additional ethical review. The consent given by the participants of MoBa cohort does not open for storage of data on an individual level in repositories or journals. Researchers who want access to MoBa dataset for replication should apply through helsedata.no. Access to MAP-19 data can be granted from the principal investigators OVE and SUJ following ethical approval of a suggested project plan for the use of data granted by NSD and REK.

## Abbreviations

C-19 Resilience Cohort: The Icelandic COVID-19 National Resilience Cohort
MAP-19 Cohort: The Norwegian COVID-19 Mental Health and Adherence Study
MoBa cohort: The Norwegian Mother, Father, and Child Cohort Study
GAD-7: The validated 7-item Generalized Anxiety Disorder Scale
PHQ-9: The validated Patient Health Questionnaire-9
BMI: Body mass index
PRs: Prevalence ratios
Cis: Confidence intervals
STROBE: The Strengthening the Reporting of Observational studies in Epidemiology
