# Supplementary material for: Long-term psychological effects of COVID-19-related quarantine: an observational study of three cohorts in Norway and Iceland
Source: BMC Med. 2025 Sep 25;23:519. doi: 10.1186/s12916-025-04349-8 (PMC12465783; doi:10.1186/s12916-025-04349-8)
Supplement: Supplementary file 1 — Additional file 1: Supplementary material. [file 12916_2025_4349_MOESM1_ESM.docx]

**Long-term psychological effects of Covid-19-related quarantine: an observational study of three cohorts in Norway and Iceland**

Li Lu^1,2#,^*, Yue Wang^3#^, Omid V. Ebrahimi^4,5#^, Qing Shen^3,6,7#^, Anna Bára Unnarsdóttir^3^, Arna Hauksdóttir^3^, Asle Hoffart^5,8^, Edda Bjork Thordardottir^3^, Ingibjörg Magnúsdóttir^3^, Jóhanna Jakobsdóttir^3^, Lill Trogstad^9^, Thor Aspelund^3^, Fang Fang^6^, Ragnhild E. Brandlistuen^2,10^, Sverre Urnes Johnson^5,8§^, Unnur Anna Valdimarsdóttir^3,6,11§^, Ole A. Andreassen^1,12§^, Helga Ask^2,8§,^*

**Affiliations:**

NORMENT Centre, Institute of Clinical Medicine, University of Oslo, Oslo, Norway.

PsychGen Centre for Genetic Epidemiology and Mental Health, Norwegian Institute of Public Health, Oslo, Norway.

Centre of Public Health Sciences, Faculty of Medicine, School of Health Sciences, University of Iceland, Reykjavik, Iceland

1. Department of Experimental Psychology, University of Oxford, Oxford, United Kingdom

Modum Bad Psychiatric Hospital and Research Center, Vikersund, Norway

Unit of Integrative Epidemiology, Institute of Environmental Medicine, Karolinska Institutet, Stockholm, Sweden

1. Clinical Research Center for Mental Disorders, Shanghai Pudong New Area Mental Health Center, Tongji University School of Medicine, Shanghai, China

Department of Psychology, University of Oslo, Oslo, Norway

Division of Infection Control, Norwegian Institute of Public Health, Oslo, Norway

The Norwegian Mother, Father and Child Cohort Study, Norwegian Institute of Public Health, Oslo, Norway.

1. Department of Epidemiology, Harvard TH Chan School of Public Health, Boston, Massachusetts, USA

NORMENT Centre, Division of Mental Health and Addiction, Oslo University Hospital, Oslo, Norway

^#^Equal contribution

^§^ Equal contribution

*Correspondence to

**Helga Ask**, Helga.Ask@fhi.no

Phone number: +47 959 49 267 (Helga)

Department of Mental Disorders, Norwegian Institute of Public Health, Oslo, Norway

**Li Lu**, liser@outlook.com

Phone number: +8613997128809 (Li)

1.NORMENT Centre, Institute of Clinical Medicine, University of Oslo, Oslo, Norway.

2.PsychGen Centre for Genetic Epidemiology and Mental Health, Norwegian Institute of Public

Health, Oslo, Norway.

Table S1 Details of exposure and outcomes data collection of each cohort

Table S2 The quarantine policies in Norway and Iceland over time

Table S3 Covariates availability of each cohort

Table S4 The prevalence of probable depression and anxiety at T_first_ and T_end_

Table S5 Characteristics by **the duration of quarantine**

Table S6 Results of crude models

Table S7 Trend analyses of specific models among the three cohorts

Table S8 Sensitivity analyses by additionally adjusted for more covariates in C19-Resilience cohort

Table S9 Sensitivity analyses by fitting the models with **MoBa data excluding participants with missing values**

Fig. S1 Prevalence ratio (PR) and 95% confidence interval (CI) of **probable depression** of participants with ***quarantine experience*** compared with those without quarantine stratified by age group, sex, COVID-19 infection and history of psychiatric disorder overall and in each cohort

Fig. S2 Prevalence ratio (PR) and 95% confidence interval (CI) of **probable anxiety** of participants with ***quarantine experience*** compared with those without quarantine stratified by age group, sex, COVID-19 infection and history of psychiatric disorder overall and in each cohort

Table S1 Details of exposure and outcomes data collection of each cohort

| **Cohort** | **Exposure and outcomes** | | **Survey** | **Date** | |
| --- | --- | --- | --- | --- | --- |
| **C-19 resilience** | Quarantine (Have you been in quarantine due to COVID-19?);  GAD-7 / PHQ-9 | | T _first_ | Apr 2020 - Jul 2020 | |
|  | Quarantine; GAD-7 / PHQ-9 | | T_2_ | Dec 2020 - Mar 2021 | |
|  | Quarantine; GAD-7 / PHQ-9 | | T _most recent_ | May 2021 - Jul 2021 | |
|  |  | |  |  | |
| **MAP-19** | Quarantine (How many times have you been in quarantine due to COVID-19?);  GAD-7 / PHQ-9 | | T _first_ | Mar 31- Apr 7, 2020 | |
|  | Quarantine; GAD-7 / PHQ-9 | | T_2_ | Jun 22 - Jul 13, 2020 | |
|  | Quarantine; GAD-7 / PHQ-9 | | T_3_ | Nov 19 - Dec 2, 2020 | |
|  | Quarantine; GAD-7 / PHQ-9 | | T_4_ | Jan 23 - Feb 2, 2021 | |
|  | Quarantine; GAD-7 / PHQ-9 | | T_5_ | May 08 - May 25, 2021 | |
|  | Quarantine; GAD-7 / PHQ-9 | | T_6_ | Jul 04 - Aug 01, 2021 | |
|  | Quarantine; GAD-7 / PHQ-9 | | T_7_ | Oct 24 – Nov 12, 2021 | |
|  | Quarantine; GAD-7 / PHQ-9 | | T_8_ | Jan 02 – Jan 14, 2022 | |
|  | Quarantine; GAD-7 / PHQ-9 | | T  _most recent_ | Mar 6 - Mar 27, 2022 | |
|  |  | |  |  | |
| **MoBa** | GAD-7 | | T _first_ | May 12- May 27, 2020 | |
|  | PHQ-9 | | T _first_ | Jun 10- Jun 24, 2020 | |
|  | GAD-7 | | T _most recent_ | Mar 2- Mar 17, 2021 | |
|  | Quarantine (Have you been in quarantine / isolation during the last 14 days?) | | **Biweekly** | From May 12-27, 2020 to Mar 2-17, 2021. | |
| - **The four constructed quarantine variables** | | | | | |
| **Variables name** | **Meaning** | **Categories** | | | **Rationale** |
| Quarantine experience ever | Ever reporting exposure to quarantine experience before the most recent assessment of the mental health outcomes | - 0: No. - 1: Yes. | | | Could help to provide a basis for identifying high-risk populations (e.g., those who have been quarantined) offer further targeted psychological support. |
| Duration of quarantine | How many weeks the participant had been quarantined in total before the most recent assessment of the mental health outcomes | - No quarantine. - 0-2 weeks:≤14 days. - 2-4 weeks: 15-28 days. - >4 weeks: >28 days. | | | Could help to determine whether there is a "dose-response relationship" (i.e., the longer the quarantine, the more severe the mental health problems), and provide a scientific basis for formulating reasonable quarantine policies (e.g., limits on quarantine duration). |
| Recency of quarantine | Time since the end of the most recent quarantine, time between the most recent quarantine and the time of the most recent assessment of the mental health outcomes | - No quarantine. - 0-2 weeks:≤14 days. - 2-4 weeks: 15-28 days. - >4 weeks: >28 days. | | | Could help to determine whether the short-term effects of quarantine on mental health diminish over time or persist, and provide guidance for designing the timing of psychological interventions (e.g., immediate intervention after quarantine vs. long-term follow-up support). |
| Total duration of quarantine between two measurements of the mental health outcomes | Total duration of quarantine between the first and the most recent measurements of the mental health outcomes | - No quarantine. - 0-2 weeks:≤14 days. - 2-4 weeks: 15-28 days. - >4 weeks: >28 days. | | | Could help to determine whether multiple or prolonged quarantines have additive or cumulative negative effects on mental health and provide evidence for evaluating the long-term impacts of quarantine policies and help optimize quarantine strategies. |

Note: Data of anxiety and depressive symptoms from the first and most recent measurement were used

in this study.

GAD-7: The 7-item Generalized Anxiety Disorder Scale.

PHQ-9: The 9-item Patient Health Questionnaire-9 (PHQ-9).

Table S2 The quarantine policies in Norway and Iceland over time.

| **Norway** | |  |  | **Iceland** |
| --- | --- | --- | --- | --- |
| **Date of entry into force** | **Policy in detail** |  | **Date of entry into force** | **Policy in detail** |
| March 13, 2020 | Persons arriving in Norway from countries other than Sweden or Finland must undergo quarantine for 14 days after entry into Norway. The Regulations apply to persons who have arrived in Norway after 27 February 2020. |  | February 28, 2020 | Icelanders arriving to Iceland from defined high-risk areas of infection and individuals who had been in contact with a confirmed case of COVID-19 should be in home-based quarantine for 14 days. |
| November 9, 2020 | People in entry quarantine must stay at a quarantine hotel during the 10-day quarantine period. This requirement will not apply to people who reside in Norway or own a home or holiday home in Norway. |  | March 19, 2020 | All residents of Iceland who enter the country are obligated to go into 14 days of quarantine, regardless of where they were arriving from. The same applies to those who had been in contact with a COVID-19-infected individual. Transportation crews (airlines and freighters) are exempt. |
| December 14, 2020 | The removal of the requirements to stay in a quarantine hotel for persons whose stay is for purposes other than work or an assignment and who can document that they have access to another suitable location for their quarantine stay. |  | March 25, 2020 | Icelandic authorities are taking strict measures to limit the spread of the COVID-19 disease in the country. The heaviest focus has been on testing, contact tracing and quarantine of individuals considered to be likely carriers. |
| December 29, 2020 | Travellers arriving in Norway from abroad may, at the earliest, end quarantine on day seven if they test negative for Covid-19 twice after arrival. The first test must be taken within three days of arrival, and the second, at the earliest, seven days after arrival. The new arrangement does not apply to travellers who have been in the United Kingdom during the last 14 days prior to arrival. |  | April 8, 2020 | Quarantine is mandatory when a person is possibly infected but is not yet symptomatic. |
| January 25, 2021 | The quarantine rules are being tightened across the board. There will be fewer exemptions in the quarantine rules and an increase in the testing requirements that apply to persons subject to exemptions. |  | April 24, 2020 | International arrivals to Iceland must be quarantined for 14 days from their day of arrival and temporary internal Schengen border controls will be introduced on the same day. Barring any changes in the medical and scientific advice, these rules will remain in place until at least May 15th. |
| February 23, 2021 | Anyone who is resident or has permanent residence in Norway has been exempt from the hotel quarantine requirement. |  | June 15, 2020 | Iceland has offered all international arrivals the opportunity to undergo COVID-19 testing at their point of entry as an alternative to a 14-day quarantine. The rule does not apply to minors (born 2005 and later) who are exempt from both quarantine and testing requirements. |
| March 16, 2021 | Anyone undertaking unnecessary leisure travel abroad must stay at a quarantine hotel when they return to Norway. For who will need to go into quarantine, the quarantine period is still ten days and can be shortened after seven days if negative test results are produced. |  | August 19, 2020 | All arriving passengers must choose between a 14-day quarantine and a double testing procedure along with a quarantine for 5-6 days. |
| May 16, 2021 | Any person who arrives in Norway from a country outside the EEA/Schengen area must stay at a quarantine hotel. This applies regardless of whether the trip has been necessary or not. |  | September 14, 2020 | Quarantine shortened from 14 days to 7, ending with a test. The new arrangement applies to infection precautions within Iceland, not to persons arriving at the border. |
| May 24, 2021 | The distinction between necessary and unnecessary travel is removed for entry into Norway so that travellers from the UK and countries in the EEA/Schengen area with a low rate of infection do not have to stay at a quarantine hotel. People who have been to areas with a high rate of infection must stay at a quarantine hotel, regardless of the purpose of the trip. Exemptions from the entry restrictions are introduced for foreign nationals who reside in areas that are not subject to the duty to quarantine in Norway. |  | January 15, 2021 | All passengers must undergo a PCR test upon arrival in Iceland, followed by a 5–6-day quarantine and a second screening at the end of quarantine period. This procedure will remain in place until 1 May, when cautious steps will be taken to ease restrictions, based on the epidemiological situation at passenger's point of departure. |
| June 7, 2021 | More people may quarantine in their own home. |  | February 16, 2021 | A negative PCR test is required prior to departure when travelling to Iceland. Additionally, a PCR test is mandatory at the border followed by a five-day quarantine and a second test. Vaccinated individuals and those with prior infection are exempt from the measures |
| June 10, 2021 | Exemption from travel quarantine.  People who being able to provide documentation of vaccination or of having recovered from COVID-19 using a secure and verifiable QR code solution during the past 6 months and will be exempt from travel quarantine. Children and adolescents under the age of 18 may shorten their quarantine period if they can present a negative result from a PCR test taken no sooner than 3 days after arrival. |  | April 1, 2021 | Stricter COVID-19 measures at the Icelandic border. Children and those traveling from areas that are classified as dark red or grey by the ECDC (where the 14-day incidence rate exceeds 500 or insufficient data is available), in the previous 14 days, will be required to stay in quarantine facilities for the duration of the five-day quarantine between tests. In case of a positive test, travelers will be required to stay in isolation in managed quarantine facilities. |
| August 28, 2021 | Transmission quarantine exemption extended from 6 to 12 months. |  | May 1, 2021 | Travellers from low-risk areas (green and yellow according to ECDC risk assessment) will be exempt from quarantine measures if they present a negative PCR result at the border. |
| December 15, 2021 | Among other things, a duty to quarantine for household members and corresponding close relations, with the possibility of taking a test to end quarantine early after 7 days.  Other close contacts may take a test to end quarantine early after 3 days, but must remain in quarantine during their leisure time until they receive a negative test after 7 days.  There is an exemption for personnel in critical societal functions during working hours, which includes a requirement for them to present a negative test taken on the same day that the work is to be done. |  | October 1, 2021 | Unvaccinated passengers will continue to undergo a five-day quarantine following testing, and to undergo a PCR test at the end of the quarantine period. |
| January 26, 2022 | Removal of travel quarantine requirements;  People who enter the country must get tested if they develop symptoms and must go into isolation if they receive a positive test result.  The 10-day transmission quarantine period has been replaced with daily testing for household members and corresponding close relations. |  | February 25, 2022 | All public restrictions due to the COVID-19 pandemic will be lifted, both domestically and at the border. Thereby all rules regarding limitations on social gatherings and school operations as well as the quarantine requirement for those infected by COVID-19 are removed. Additionally, no disease prevention measures will be in place at the border, regardless of whether individuals are vaccinated or unvaccinated. |

Note: Quarantine policies were summarized based on “Timeline: News from Norwegian Ministries about the Coronavirus disease Covid-19”. <https://www.regjeringen.no/en/topics/health-and-care/public-health/timeline-for-news-from-norwegian-ministries-about-the-coronavirus-disease-covid-19/id2692402/> Government of Iceland. <https://www.government.is/government/covid-19/#news>.

Table S3 Covariates availability of each cohort.

| **Available covariates** | **C-19 resilience** | **MAP-19** | **Moba** |
| --- | --- | --- | --- |
| Gender | Yes | Yes | Yes |
| Age | Yes | Yes | Yes |
| Highest level of attained education | Yes | Yes | Yes |
| BMI | Yes | Yes | Yes |
| Current Smoking | Yes | Yes | Yes |
| History of psychiatric disorder | Yes | Yes | Yes |
| Chronic medical conditions | Yes | Yes | Yes |
| COVID-19 diagnosis | Yes | Yes | Yes |
| Response period | Yes | Yes | Yes |
| Living condition before the most recent measurement of mental health outcomes * | NA | Yes | Yes |
| Current living condition * | NA | Yes | Yes |
| Current quarantine | Yes | Yes | Yes |
| Children in need of care | Yes | NA | NA |
| Binge drinking | Yes | NA | NA |
| Personal monthy income at baseline | Yes | NA | NA |
| Employment status at baseline | Yes | NA | NA |

NA: not applicable.

* Not adjusted for in MoBa for the longitudinal analysis since no enough participants in m subgroups.

Table S4 The prevalence of probable depression and anxiety at T_first_ and T_end_

| **Cohorts** | **Time point** | **Probable depression**  Prevalence (95% CI) | **Probable anxiety**  Prevalence (95% CI) |
| --- | --- | --- | --- |
| **C-19 resilience (N=10431) ^#^** | T_first_ | 15.5% (14.8%-16.2%) | 10.4% (9.8%-11.0%) |
| **C-19 resilience**  **(longitudinal design; N=6297)*** | T_first_ | 13.3% (12.4%-14.1%) | 9.0% (8.3%-9.8%) |
|  | T_most recent_ | 13.5% (12.7%-14.4%) | 8.4% (7.7%-9.1%) |
| **MAP-19 (N=2963)^#^** | T_first_ | 29.5% (27.8%-31.3%) | 17.6% (16.3%-19.0%) |
| **MAP-19**  **(longitudinal design; N=1754)*** | T_first_ | 28.7% (26.6%-30.8%) | 18.0% (16.2%-19.8%) |
|  | T_most recent_ | 23.5% (21.5%-25.5%) | 14.5% (12.8%-16.1%) |
| **MoBa (N=81041/91950)^#^** | T_first_ | 4.9% (4.7%-5.0%) | 3.6% (3.5%-3.7%) |
| **Moba**  **(longitudinal design; N=56260)*** | T_first_ | **-** | 2.3% (2.2%-2.5%) |
|  | T_most recent_ | **-** | 3.4% (3.2%-3.5%) |

*: The prevalence among individuals with repeated data on mental health.

**^#^**: The prevalence among the whole participants of each cohort.

95% CI: 95% confidence interval.

Table S5 Characteristics by **the duration of quarantine**

|  | **Iceland** | | | | | **Norway** | | | | | | | | | | | | | | |
| --- | --- | --- | --- | --- | --- | --- | --- | --- | --- | --- | --- | --- | --- | --- | --- | --- | --- | --- | --- | --- |
|  | **C19-Resilience**  **N=10199** | | | | | **MAP-19**  **N=2644** | | | | | **MoBa**  **Depression (PHQ-9)**  **N=91950** | | | | | **MoBa**  **Anxiety (GAD-7)**  **N=81041** | | | | |
|  | No quarantine | < = 2 weeks | 2-4 weeks | > 4 weeks | Total | No quarantine | < = 2 weeks | 2-4 weeks | > 4 weeks | Total | No quarantine | < = 2 weeks | 2-4 weeks | > 4 weeks | Total | No quarantine | < = 2 weeks | 2-4 weeks | > 4 weeks | Total |
|  | n (%) | n (%) | n (%) | n (%) | n (%) | n (%) | n (%) | n (%) | n (%) | n (%) | n (%) | n (%) | n (%) | n (%) | n (%) | n (%) | n (%) | n (%) | n (%) | n (%) |
|  | 6038  (59.2%) | 2919  (28.6%) | 508  (5.0%) | 734  (7.2%) | 10199 | 1018  (38.5%) | 774  (28.1%) | 733  (27.7%) | 119  (4.5%) | 2644 | 78677 (85.6%) | 10664 (11.6%) | 2047 (2.2%) | 562 (0.6%) | 91950 | 49254 (60.8%) | 17858 (22.0%) | 8422 (10.4%) | 5507 (6.8%) | 81041 |
| **Gender** |  |  |  |  |  |  |  |  |  |  |  |  |  |  |  |  |  |  |  |  |
| Female | 4037 (66.9%) | 2042 (70.0%) | 338 (66.5%) | 533 (72.6%) | 6950 (68.1%) | 773 (75.9%) | 599 (77.4%) | 573 (78.2%) | 98 (82.4%) | 2043  (77.3%) | 47041 (59.8%) | 6627 (62.1%) | 1347 (65.8%) | 396 (70.5%) | 55411 (60.3%) | 28685 (58.2%) | 11426 (64.0%) | 5706 (67.8%) | 3919 (71.2%) | 49736 (61.4%) |
| Male | 1944 (32.2%) | 849 (29.1%) | 161 (31.7%) | 191 (26.0%) | 3145 (30.8%) | 243 (23.9%) | 173 (22.4%) | 158 (21.6%) | 21 (17.6%) | 595  (22.5%) | 31636 (40.2%) | 4037 (37.9%) | 700 (34.2%) | 166 (29.5%) | 36539 (39.7%) | 20569 (41.8%) | 6432 (36.0%) | 2716 (32.2%) | 1588 (28.8%) | 31305 (38.6%) |
| Other | 6 (0.1%) | 3 (0.1%) | 0 (0%) | 4 (0.5%) | 13 (0.1%) | 2 (0.2%) | 2 (0.3%) | 2 (0.3%) | 0 (0%) | 6  (0.2%) | 0 | 0 | 0 | 0 | 0 | 0 | 0 | 0 | 0 | 0 |
| Missing | 51 (0.8%) | 25 (0.9%) | 9 (1.8%) | 6 (0.8%) | 91 (0.9%) | 0 (0%) | 0 (0%) | 0 (0%) | 0 (0%) | 0 (0%) | 0 | 0 | 0 | 0 | 0 | 0 | 0 | 0 | 0 | 0 |
| **Age** |  |  |  |  |  |  |  |  |  |  |  |  |  |  |  |  |  |  |  |  |
| Mean. years (SD) | 56.7 (13.0) | 53.7 (13.5) | 55.0 (13.8) | 58.8 (14.6) | 55.9 (13.4) | 39.7 (14.6) | 36.7 (13.1) | 37.6 (13.8) | 36.2 (12.4) | 38.1 (13.9) | 47.1 (5.21) | 46.9 (5.24) | 46.9 (5.46) | 46.8 (5.41) | 47.0 (5.22) | 47.5 (5.24) | 46.9 (5.11) | 46.5 (5.10) | 46.2 (5.22) | 47.2 (5.21) |
| < 35 years | 410 (6.8%) | 294 (10.1%) | 49 (9.6%) | 62 (8.4%) | 815 (8.0%) | 460 (45.2%) | 413 (53.4%) | 371 (50.6%) | 64 (53.8%) | 1467 (49.5%) | 341 (0.4%) | 68 (0.6%) | 16 (0.8%) | 4 (0.7%) | 429 (0.5%) | 143 (0.3%) | 77 (0.4%) | 40 (0.5%) | 41 (0.7%) | 301 (0.4%) |
| 35-44 years | 600 (9.9%) | 439 (15.0%) | 69 (13.6%) | 75 (10.2%) | 1183 (11.6%) | 215 (21.1%) | 169 (21.8%) | 149 (20.3%) | 29 (24.4%) | 624 (21.1%) | 23455 (29.8%) | 3273 (30.7%) | 632 (30.9%) | 172 (30.6%) | 27532 (29.9%) | 13308 (27.0%) | 5558 (31.1%) | 2789 (33.1%) | 1939 (35.2%) | 23594 (29.1%) |
| 45-54 years | 1407 (23.3%) | 690 (23.6%) | 105 (20.7%) | 108 (14.7%) | 2310 (22.6%) | 158 (15.5%) | 100 (12.9%) | 117 (16.0%) | 13 (10.9%) | 433 (14.6%) | 46524 (59.1%) | 6197 (58.1%) | 1161 (56.7%) | 323 (57.5%) | 54205 (59.0%) | 29937 (60.8%) | 10513 (58.9%) | 4821 (57.2%) | 3051 (55.4%) | 48322 (59.6%) |
| 55-64 years | 1779 (29.5%) | 806 (27.6%) | 134 (26.4%) | 175 (23.8%) | 2894 (28.4%) | 111 (10.9%) | 61 (7.9%) | 60 (8.2%) | 9 (7.6%) | 276 (9.3%) | 5492 (7.0%) | 684 (6.4%) | 142 (6.9%) | 32 (5.7%) | 6350 (6.9%) | 3931 (8.0%) | 1130 (6.3%) | 471 (5.6%) | 275 (5.0%) | 5807 (7.2%) |
| 65 years or more | 1842 (30.5%) | 690 (23.6%) | 151 (29.7%) | 314 (42.8%) | 2997 (29.4%) | 74 (7.3%) | 31 (4.0%) | 36 (4.9%) | 4 (3.4%) | 163 (5.5%) | 235 (0.3%) | 29 (0.3%) | 6 (0.3%) | 2 (0.4%) | 272 (0.3%) | 204 (0.4%) | 41 (0.2%) | 18 (0.2%) | 19 (0.3%) | 282 (0.3%) |
| Missing | 0 (0%) | 0 (0%) | 0 (0%) | 0 (0%) | 0 (0%) | 0 (0%) | 0 (0%) | 0 (0%) | 0 (0%) | 0 (0%) | 2630 (3.3%) | 413 (3.9%) | 90 (4.4%) | 29 (5.2%) | 3162 (3.4%) | 1731 (3.5%) | 539 (3.0%) | 283 (3.4%) | 182 (3.3%) | 2735 (3.4%) |
| **Education** |  |  |  |  |  |  |  |  |  |  |  |  |  |  |  |  |  |  |  |  |
| No formal education | 0 (0%) | 0 (0%) | 0 (0%) | 0 (0%) | 0 (0%) | 1 (0.1%) | 1 (0.1%) | 2 (0.3%) | 0 (0%) | 4 (0.1%) | 0 | 0 | 0 | 0 | 0 | 0 | 0 | 0 | 0 | 0 |
| Compulsory | 840 (13.9%) | 301 (10.3%) | 59 (11.6%) | 120 (16.3%) | 1320 (12.9%) | 55 (5.4%) | 36 (4.7%) | 24 (3.3%) | 4 (3.4%) | 136 (4.6%) | 1415 (1.8%) | 183 (1.7%) | 50 (2.4%) | 25 (4.4%) | 1673 (1.8%) | 898 (1.8%) | 269 (1.5%) | 139 (1.7%) | 97 (1.8%) | 1403 (1.7%) |
| Upper secondary, vocational or other | 1885 (31.2%) | 808 (27.7%) | 161 (31.7%) | 230 (31.3%) | 3084 (30.2%) | 367 (36.1%) | 292 (37.7%) | 256 (34.9%) | 41 (34.5%) | 1061 (35.8%) | 22350 (28.4%) | 2730 (25.6%) | 610 (29.8%) | 159 (28.3%) | 25849 (28.1%) | 14193 (28.8%) | 4535 (25.4%) | 2096 (24.9%) | 1341 (24.4%) | 22165 (27.4%) |
| Bachelor's/ diploma university degree | 1918 (31.8%) | 967 (33.1%) | 161 (31.7%) | 217 (29.6%) | 3263 (32.0%) | 595 (58.4%) | 445 (57.5%) | 451 (61.5%) | 74 (62.2%) | 1762 (59.5%) | 27538 (35.0%) | 3816 (35.8%) | 712 (34.8%) | 182 (32.4%) | 32248 (35.1%) | 17086 (34.7%) | 6635 (37.2%) | 3149 (37.4%) | 2055 (37.3%) | 28925 (35.7%) |
| Master's or Ph.D. | 1318 (21.8%) | 810 (27.7%) | 116 (22.8%) | 157 (21.4%) | 2401 (23.5%) | - | - | - | - | - | 22929 (29.1%) | 3216 (30.2%) | 530 (25.9%) | 141 (25.1%) | 26816 (29.2%) | 14277 (29.0%) | 5461 (30.6%) | 2543 (30.2%) | 1673 (30.4%) | 23954 (29.6%) |
| Missing | 77 (1.3%) | 33 (1.1%) | 11 (2.2%) | 10 (1.4%) | 131 (1.3%) | - | - | - | - | - | 4445 (5.6%) | 719 (6.7%) | 145 (7.1%) | 55 (9.8%) | 5364 (5.8%) | 2800 (5.7%) | 958 (5.4%) | 495 (5.9%) | 341 (6.2%) | 4594 (5.7%) |
| **BMI (kg/m^2)** |  |  |  |  |  |  |  |  |  |  |  |  |  |  |  |  |  |  |  |  |
| < 25, Normal or low weight | 1647 (27.3%) | 866 (29.7%) | 145 (28.5%) | 185 (25.2%) | 2843 (27.9%) | 427 (41.9%) | 356 (46.0%) | 339 (46.2%) | 55 (46.2%) | 1317 (44.4%) | 25980 (33.0%) | 3343 (31.3%) | 564 (27.6%) | 179 (31.9%) | 30066 (32.7%) | 18097 (36.7%) | 6622 (37.1%) | 3157 (37.5%) | 2011 (36.5%) | 29887 (36.9%) |
| 25-30, Overweight | 2321 (38.4%) | 1084 (37.1%) | 187 (36.8%) | 252 (34.3%) | 3844 (37.7%) | 381 (37.4%) | 244 (31.5%) | 227 (31.0%) | 40 (33.6%) | 984 (33.2%) | 21879 (27.8%) | 3000 (28.1%) | 548 (26.8%) | 103 (18.3%) | 25530 (27.8%) | 15269 (31.0%) | 5623 (31.5%) | 2668 (31.7%) | 1730 (31.4%) | 25290 (31.2%) |
| > 30, Obese | 1868 (30.9%) | 897 (30.7%) | 159 (31.3%) | 272 (37.1%) | 3196 (31.3%) | 210 (20.6%) | 174 (22.5%) | 167 (22.8%) | 24 (20.2%) | 662 (22.3%) | 9850 (12.5%) | 1408 (13.2%) | 325 (15.9%) | 114 (20.3%) | 11697 (12.7%) | 6685 (13.6%) | 2691 (15.1%) | 1337 (15.9%) | 999 (18.1%) | 11712 (14.5%) |
| Missing | 202 (3.3%) | 72 (2.5%) | 17 (3.3%) | 25 (3.4%) | 316 (3.1%) | 0 (0%) | 0 (0%) | 0 (0%) | 0 (0%) | 0 (0%) | 20968 (26.7%) | 2913 (27.3%) | 610 (29.8%) | 166 (29.5%) | 24657 (26.8%) | 9203 (18.7%) | 2922 (16.3%) | 1260 (15.0%) | 767 (13.9%) | 14152 (17.5%) |
| **Current smoking status** |  |  |  |  |  |  |  |  |  |  |  |  |  |  |  |  |  |  |  |  |
| No | 5135 (85.0%) | 2446 (83.8%) | 430 (84.6%) | 594 (80.9%) | 8605 (84.4%) | 941 (92.4%) | 724 (93.5%) | 674 (92.0%) | 117 (98.3%) | 2752 (92.9%) | 68901 (87.6%) | 9238 (86.6%) | 1738 (84.9%) | 461 (82.0%) | 80338 (87.4%) | 41009 (83.3%) | 15237 (85.3%) | 7207 (85.6%) | 4636 (84.2%) | 68089 (84.0%) |
| Yes | 798 (13.2%) | 425 (14.6%) | 65 (12.8%) | 133 (18.1%) | 1421 (13.9%) | 77 (7.6%) | 50 (6.5%) | 59 (8.0%) | 2 (1.7%) | 211 (7.1%) | 7117 (9.0%) | 1010 (9.5%) | 218 (10.6%) | 72 (12.8%) | 8417 (9.2%) | 3968 (8.1%) | 1505 (8.4%) | 745 (8.8%) | 597 (10.8%) | 6815 (8.4%) |
| Missing | 105 (1.7%) | 48 (1.6%) | 13 (2.6%) | 7 (1.0%) | 173 (1.7%) | 0 (0%) | 0 (0%) | 0 (0%) | 0 (0%) | 0 (0%) | 2659 (3.4%) | 416 (3.9%) | 91 (4.4%) | 29 (5.2%) | 3195 (3.5%) | 4277 (8.7%) | 1116 (6.2%) | 470 (5.6%) | 274 (5.0%) | 6137 (7.6%) |
| **History of psychiatric disorder** |  |  |  |  |  |  |  |  |  |  |  |  |  |  |  |  |  |  |  |  |
| No | 4352 (72.1%) | 2005 (68.7%) | 350 (68.9%) | 449 (61.2%) | 7156 (70.2%) | 781 (76.7%) | 607 (78.4%) | 553 (75.4%) | 94 (79.0%) | 2280 (76.9%) | 63794 (81.1%) | 8389 (78.7% | 1552 (75.8%) | 400 (71.2%) | 74135 (80.6%) | 40369 (82.0%) | 14321 (80.2%) | 6608 (78.5%) | 4120 (74.8%) | 65418 (80.7%) |
| Yes | 1560 (25.8%) | 851 (29.2%) | 141 (27.8%) | 268 (36.5%) | 2820 (27.6%) | 237 (23.3%) | 167 (21.6%) | 180 (24.6%) | 25 (21.0%) | 683 (23.1%) | 12179 (15.5%) | 1850 (17.3%) | 402 (19.6%) | 132 (23.5%) | 14563 (15.8%) | 7118 (14.5%) | 2979 (16.7%) | 1522 (18.1%) | 1200 (21.8%) | 12819 (15.8%) |
| Missing | 126 (2.1%) | 63 (2.2%) | 17 (3.3%) | 17 (2.3%) | 223 (2.2%) | 0 (0%) | 0 (0%) | 0 (0%) | 0 (0%) | 0 (0%) | 2704 (3.4%) | 425 (4.0%) | 93 (4.5%) | 30 (5.3%) | 3252 (3.5%) | 1767 (3.6%) | 558 (3.1%) | 292 (3.5%) | 187 (3.4%) | 2804 (3.5%) |
| **Pre-existing comorbidity** |  |  |  |  |  |  |  |  |  |  |  |  |  |  |  |  |  |  |  |  |
| No | 3302 (54.7%) | 1751 (60.0%) | 272 (53.5%) | 265 (36.1%) | 5590 (54.8%) | 645 (63.4%) | 522 (67.4%) | 505 (68.9%) | 80 (67.2%) | 1969 (66.5%) | 63189 (80.3%) | 8261 (77.5%) | 1502 (73.4%) | 371 (66.0%) | 73323 (79.7%) | 38375 (77.9%) | 14110 (79.0%) | 6546 (77.7%) | 4159 (75.5%) | 63190 (78.0%) |
| One comorbidity* | 1879 (31.1%) | 822 (28.2%) | 152 (29.9%) | 232 (31.6%) | 3085 (30.2%) | 373 (36.6%) | 252 (32.6%) | 228 (31.1%) | 39 (32.8%) | 994 (33.5%) | 11348 (14.4%) | 1700 (15.9%) | 372 (18.2%) | 123 (21.9%) | 13543 (14.7%) | 6760 (13.7%) | 2615 (14.6%) | 1329 (15.8%) | 964 (17.5%) | 11668 (14.4%) |
| Two or more comorbidities | 771 (12.8%) | 304 (10.4%) | 72 (14.2%) | 231 (31.5%) | 1378 (13.5%) | - | - | - | - | - | 1509 (1.9%) | 290 (2.7%) | 83 (4.1%) | 39 (6.9%) | 1921 (2.1%) | 922 (1.9%) | 381 (2.1%) | 187 (2.2%) | 177 (3.2%) | 1667 (2.1%) |
| Missing | 86 (1.4%) | 42 (1.4%) | 12 (2.4%) | 6 (0.8%) | 146 (1.4%) | 0 (0%) | 0 (0%) | 0 (0%) | 0 (0%) | 0 (0%) | 2631 (3.3%) | 413 (3.9%) | 90 (4.4%) | 29 (5.2%) | 3163 (3.4%) | 3197 (6.5%) | 752 (4.2%) | 360 (4.3%) | 207 (3.8%) | 4516 (5.6%) |
| **Participant's COVID-19 status** |  |  |  |  |  |  |  |  |  |  |  |  |  |  |  |  |  |  |  |  |
| Not infected | 5951 (98.6%) | 2647 (90.7%) | 398 (78.3%) | 664 (90.5%) | 9660 (94.7%) | 603 (59.2%) | 391 (50.5%) | 312 (42.6%) | 50 (42.0%) | 1511 (51.0%) | 78661  (100) | 10609  (99.5) | 1951  (95.3) | 522  (92.9) | 91743  (99.8) | 49180  (99.8) | 17608  (98.6) | 8045  (95.5) | 5201  (94.4) | 80034  (98.8) |
| Infected | 86 (1.4%) | 271 (9.3%) | 107 (21.1%) | 70 (9.5%) | 534 (5.2%) | 415 (40.8%) | 383 (49.5%) | 421 (57.4%) | 69 (58.0%) | 1452 (49.0%) | 16  (0.0) | 55  (0.5) | 96  (4.7) | 40  (7.1) | 207  (0.2) | 74  (0.2) | 250  (1.4) | 377  (4.5) | 306  (5.6) | 1007  (1.2) |
| Missing | 1 (0.0%) | 1 (0.0%) | 3 (0.6%) | 0 (0%) | 5 (0.0%) | 0 (0%) | 0 (0%) | 0 (0%) | 0 (0%) | 0 (0%) | 0 (0%) | 0 (0%) | 0 (0%) | 0 (0%) | 0 (0%) | 0 (0%) | 0 (0%) | 0 (0%) | 0 (0%) | 0 (0%) |
| **Quarantine during the most recent measurement** |  |  |  |  |  |  |  |  |  |  |  |  |  |  |  |  |  |  |  |  |
| No | 6038 (100%) | 2832 (97.0%) | 498 (98.0%) | 640 (87.2%) | 10008 (98.1%) | 712 (69.9%) | 251 (32.4%) | 201 (27.4%) | 51 (42.9%) | 1215 (46.0%) | 78211 (99.4%) | 10457 (98.1%) | 1977 (96.6%) | 494 (87.9%) | 91139 (99.1%) | 47276 (96.0%) | 16646 (93.2%) | 7755 (92.1%) | 4840 (87.9%) | 76517 (94.4%) |
| Yes | 0 (0%) | 37 (1.3%) | 6 (1.2%) | 53 (7.2%) | 96 (0.9%) | 306 (30.1%) | 166 (21.4%) | 179 (24.4%) | 6 (5.0%) | 657 (24.8%) | 366 (0.5%) | 197 (1.8%) | 67 (3.3%) | 68 (12.1%) | 698 (0.8%) | 1924 (3.9%) | 1199 (6.7%) | 663 (7.9%) | 666 (12.1%) | 4452 (5.5%) |
| Missing | 0 (0%) | 50 (1.7%) | 4 (0.8%) | 41 (5.6%) | 95 (0.9%) | 0 (0%) | 357 (46.1%) | 353 (48.2%) | 62 (52.1%) | 772 (29.2%) | 100 (0.1%) | 10 (0.1%) | 3 (0.1%) | 0 (0%) | 113 (0.1%) | 54 (0.1%) | 13 (0.1%) | 4 (0%) | 1 (0%) | 72 (0.1%) |
| **Living condition before the latest measurement** |  |  |  |  |  |  |  |  |  |  |  |  |  |  |  |  |  |  |  |  |
| Living with other people | - | - | - | - | - | 318 (31.2%) | 175 (22.6%) | 193 (26.3%) | 29 (24.4%) | 715 (27.0%) | 741 (0.9%) | 135 (1.3%) | 42 (2.1%) | 27 (4.8%) | 945 (1.0%) | 490 (1.0%) | 155 (0.9%) | 86 (1.0%) | 91 (1.7%) | 822 (1.0%) |
| Living alone | - | - | - | - | - | 700 (68.8%) | 595 (76.9%) | 540 (73.7%) | 90 (75.6%) | 1925  (72.8%) | 73469 (93.4%) | 10333 (96.9%) | 1963 (95.9%) | 526 (93.6%) | 86291 (93.8%) | 44433 (90.2%) | 16759 (93.8%) | 7933 (94.2%) | 5229 (95.0%) | 74354 (91.7%) |
| Missing | 6038  (100%) | 2919  (100%) | 508  (100%) | 734  (100%) | 10199(100%) | 0 (0%) | 4 (0.5%) | 0 (0%) | 0 (0%) | 4 (0.2%) | 4467 (5.7%) | 196 (1.8%) | 42 (2.1%) | 9 (1.6%) | 4714 (5.1%) | 4331 (8.8%) | 944 (5.3%) | 403 (4.8%) | 187 (3.4%) | 5865 (7.2%) |
| **Living condition at the latest measurement** |  |  |  |  |  |  |  |  |  |  |  |  |  |  |  |  |  |  |  |  |
| Living with other people | - | - | - | - | - | 320 (31.4%) | 183 (23.6%) | 200 (27.3%) | 30 (25.2%) | 733  (27.7%) | 608 (0.8%) | 95 (0.9%) | 30 (1.5%) | 21 (3.7%) | 754 (0.8%) | 386 (0.8%) | 114 (0.6%) | 58 (0.7%) | 59 (1.1%) | 617 (0.8%) |
| Living alone | - | - | - | - | - | 698 (68.6%) | 591 (76.4%) | 533 (72.7%) | 89 (74.8%) | 1911 (72.6%) | 68204 (86.7%) | 9026 (84.6%) | 1696 (82.9%) | 459 (81.7%) | 79385 (86.3%) | 37282 (75.7%) | 13469 (75.4) | 6378 (75.7%) | 4184 (76.0%) | 61313 (75.7%) |
| Missing | 6038  (100%) | 2919  (100%) | 508  (100%) | 734  (100%) | 10199(100%) | 0 (0%) | 0 (0%) | 0 (0%) | 0 (0%) | 0 (0%) | 9865 (12.5%) | 1543 (14.5%) | 321 (15.7%) | 82 (14.6%) | 11811 (12.8%) | 11586 (23.5%) | 4275 (23.9%) | 1986 (23.6%) | 1264 (23.0%) | 19111 (23.6%) |

*: For MAP-19, only data of one comorbidity or more was available.

Table S6 Results of crude models**.**

|  |  |  | **C-19 Resilience** | | |  | **MAP-19** | | |  | **MoBa** | | |  | **Overall** | | |
| --- | --- | --- | --- | --- | --- | --- | --- | --- | --- | --- | --- | --- | --- | --- | --- | --- | --- |
| Probable depression |  |  | PR _crude_ | 95% CI | |  | PR _crude_ | 95% CI | |  | PR _crude_ | 95% CI | |  | PR _crude_ | 95% CI | |
|  |  |  |  | Lower | Upper |  |  | Lower | Upper |  |  | Lower | Upper |  |  | Lower | Upper |
|  | Quarantine experience (Ref: no) | Yes | 1.30 | 1.19 | 1.43 |  | 1.08 | 0.99 | 1.17 |  | 1.62 | 1.51 | 1.74 |  | 1.32 | 1.09 | 1.59 |
|  | Duration of quarantine (Ref: no) | < = 2 weeks | 1.14 | 1.02 | 1.27 |  | 1.12 | 0.96 | 1.32 |  | 1.44 | 1.33 | 1.56 |  | 1.23 | 0.87 | 1.74 |
|  |  | 2-4 weeks | 1.35 | 1.11 | 1.65 |  | 1.22 | 1.04 | 1.44 |  | 2.05 | 1.79 | 2.36 |  | 1.51 | 1.06 | 2.14 |
|  |  | > 4 weeks | 1.93 | 1.68 | 2.23 |  | 1.41 | 1.06 | 1.86 |  | 3.50 | 2.88 | 4.25 |  | 2.14 | 1.50 | 3.07 |
|  | Time since the most recent quarantine (Ref: no) | > 4 weeks | 1.25 | 1.14 | 1.38 |  | 1.11 | 0.96 | 1.28 |  | 1.63 | 1.49 | 1.77 |  | 1.31 | 0.98 | 1.77 |
|  |  | 2-4 weeks | 1.24 | 0.88 | 1.77 |  | 1.44 | 1.08 | 1.93 |  | 2.76 | 1.44 | 2.17 |  | 1.76 | 1.26 | 2.46 |
|  |  | < = 2 weeks | 1.97 | 1.55 | 2.50 |  | 1.62 | 1.21 | 2.18 |  | 2.26 | 1.82 | 2.81 |  | 1.95 | 1.41 | 2.69 |
|  | Longitudinal model (Ref: no) | < = 2 weeks | 1.14 | 1.00 | 1.30 |  | 1.16 | 1.001 | 1.34 |  | **-** | - | - |  | 1.15 | 0.93 | 1.42 |
|  |  | 2-4 weeks | 1.65 | 1.15 | 2.37 |  | 1.11 | 0.96 | 1.29 |  | **-** | - | - |  | 1.26 | 0.98 | 1.62 |
|  |  | > 4 weeks | 1.99 | 1.52 | 2.60 |  | 1.43 | 1.11 | 1.84 |  | **-** | - | - |  | 1.68 | 1.28 | 2.19 |
| Probable anxiety | Quarantine experience (Ref: no) | Yes | 1.40 | 1.24 | 1.58 |  | 1.09 | 0.95 | 1.26 |  | 1.51 | 1.41 | 1.62 |  | 1.34 | 1.17 | 1.54 |
|  | Duration of quarantine | < = 2 weeks | 1.21 | 1.05 | 1.40 |  | 1.06 | 0.84 | 1.34 |  | 1.26 | 1.16 | 1.38 |  | 1.21 | 1.08 | 1.35 |
|  |  | 2-4 weeks | 1.46 | 1.13 | 1.88 |  | 1.22 | 0.97 | 1.54 |  | 1.49 | 1.33 | 1.67 |  | 1.41 | 1.24 | 1.61 |
|  |  | > 4 weeks | 2.11 | 1.75 | 2.53 |  | 1.12 | 0.69 | 1.82 |  | 2.36 | 2.12 | 2.63 |  | 2.15 | 1.88 | 2.46 |
|  | Time since the most recent quarantine | > 4 weeks | 1.35 | 1.19 | 1.53 |  | 1.06 | 0.86 | 1.31 |  | 1.56 | 1.44 | 1.68 |  | 1.33 | 1.06 | 1.67 |
|  |  | 2-4 weeks | 1.15 | 0.71 | 1.88 |  | 1.56 | 1.04 | 2.33 |  | 1.97 | 1.61 | 2.41 |  | 1.63 | 1.21 | 2.18 |
|  |  | < = 2 weeks | 1.95 | 1.40 | 2.72 |  | 1.80 | 1.17 | 2.76 |  | 1.28 | 1.13 | 1.45 |  | 1.57 | 1.19 | 2.05 |
|  | Longitudinal model (Ref: no) | < = 2 weeks | 1.21 | 1.03 | 1.42 |  | 1.09 | 0.90 | 1.33 |  | 1.21 | 1.09 | 1.34 |  | 1.17 | 0.99 | 1.39 |
|  |  | 2-4 weeks | 1.99 | 1.33 | 2.98 |  | 1.16 | 0.96 | 1.40 |  | 1.53 | 1.35 | 1.74 |  | 1.44 | 1.19 | 1.75 |
|  |  | > 4 weeks | 1.52 | 1.02 | 2.27 |  | 1.32 | 0.93 | 1.88 |  | 2.01 | 1.72 | 2.36 |  | 1.70 | 1.37 | 2.12 |

PR: Prevalence ratio; 95% CI: 95% confidence interval

Table S7 Trend analyses of specific models among the three cohorts

|  |  | Estimate | Standard error | Statistic | R^2^ | P value |
| --- | --- | --- | --- | --- | --- | --- |
| Probable depression | Duration of quarantine | 0.294 | 0.048 | 6.174 | 0.993 | **<0.001** |
|  | Time since the most recent quarantine | -0.190 | 0.059 | -3.236 | 1 | **0.001** |
|  | Longitudinal model | 0.290 | 0.117 | 2.475 | 0.534 | **0.013** |
|  |  |  |  |  |  |  |
| Probable anxiety | Duration of quarantine | 0.318 | 0.043 | 7.451 | 1 | **<0.001** |
|  | Time since the most recent quarantine | -0.081 | 0.125 | -0.654 | 0 | 0.513 |
|  | Longitudinal model | 0.258 | 0.057 | 4.498 | 0.999 | **<0.001** |

Table S8 Sensitivity analyses **by additionally adjusted for *more covariates* in C19-Resilience cohort.**

|  |  |  | Step 1 | | | | | |  | Step 2 | | | | | |
| --- | --- | --- | --- | --- | --- | --- | --- | --- | --- | --- | --- | --- | --- | --- | --- |
|  |  |  | Estimate | Standard error | Statistic | P value | 95% CI | |  | Estimate | Standard error | Statistic | P value | 95% CI | |
|  |  |  |  |  |  |  | Lower | Upper |  |  |  |  |  | Lower | Upper |
| Probable depression | Duration of quarantine | < = 2 weeks | 1.00 | 0.05 | -0.04 | 0.97 | 0.90 | 1.10 |  | 1.00 | 0.05 | -0.01 | 0.99 | 0.9 | 1.11 |
|  |  | 2-4 weeks | 1.21 | 0.10 | 1.94 | 0.05 | 1.00 | 1.46 |  | 1.18 | 0.10 | 1.71 | 0.09 | 0.98 | 1.43 |
|  |  | > 4 weeks | 1.47 | 0.07 | 5.28 | **<0.001** | 1.28 | 1.70 |  | 1.40 | 0.07 | 4.53 | **<0.001** | 1.21 | 1.62 |
|  |  |  |  |  |  |  |  |  |  |  |  |  |  |  |  |
|  | Time since the most recent quarantine | > 4 weeks | 1.08 | 0.05 | 1.54 | 0.12 | 0.98 | 1.18 |  | 1.07 | 0.05 | 1.39 | 0.16 | 0.97 | 1.17 |
|  |  | 2-4 weeks | 1.13 | 0.16 | 0.75 | 0.46 | 0.82 | 1.55 |  | 1.13 | 0.15 | 0.79 | 0.43 | 0.84 | 1.52 |
|  |  | < = 2 weeks | 1.62 | 0.12 | 3.88 | **<0.001** | 1.27 | 2.06 |  | 1.59 | 0.13 | 3.63 | **<0.001** | 1.24 | 2.04 |
|  |  |  |  |  |  |  |  |  |  |  |  |  |  |  |  |
| Probable anxiety | Duration of quarantine | < = 2 weeks | 1.02 | 0.07 | 0.28 | 0.78 | 0.89 | 1.17 |  | 1.02 | 0.07 | 0.33 | 0.74 | 0.89 | 1.17 |
|  |  | 2-4 weeks | 1.32 | 0.13 | 2.19 | **0.03** | 1.03 | 1.69 |  | 1.28 | 0.12 | 2.00 | 0.05 | 1.01 | 1.64 |
|  |  | > 4 weeks | 1.69 | 0.09 | 5.67 | **<0.001** | 1.41 | 2.03 |  | 1.60 | 0.09 | 5.01 | **<0.001** | 1.33 | 1.92 |
|  |  |  |  |  |  |  |  |  |  |  |  |  |  |  |  |
|  | Time since the most recent quarantine | > 4 weeks | 1.14 | 0.06 | 2.05 | **0.04** | 1.01 | 1.29 |  | 1.12 | 0.06 | 1.8 | 0.07 | 0.99 | 1.27 |
|  |  | 2-4 weeks | 0.99 | 0.24 | -0.06 | 0.95 | 0.62 | 1.57 |  | 1.00 | 0.23 | -0.01 | 0.99 | 0.64 | 1.57 |
|  |  | < = 2 weeks | 1.58 | 0.17 | 2.65 | **0.01** | 1.13 | 2.21 |  | 1.53 | 0.17 | 2.52 | **0.01** | 1.10 | 2.13 |

Note: Step 1, Adjusted for age group, gender, education, BMI, current smoking, history of psychiatric disorder, chronic medical conditions, COVID-19 diagnosis, current quarantine + *number of children in care* and *binge* *drinking.*

Step 2, Adjusted for covariates in step 1 + personal monthly income, and employment status at baseline.

Table S9 Sensitivity analyses by fitting the models with **MoBa data excluding participants with missing values.**

|  |  |  | Estimate | Standard error | Statistic | P value | 95% CI | |
| --- | --- | --- | --- | --- | --- | --- | --- | --- |
|  |  |  |  |  |  |  | Lower | Upper |
| Probable depression | Duration of quarantine (N=53656) | < = 2 weeks | 1.20 | 0.06 | 10.5 | **0.001** | 1.08 | 1.34 |
|  |  | 2-4 weeks | 1.34 | 0.07 | 18.0 | **<0.001** | 1.17 | 1.54 |
|  |  | > 4 weeks | 1.88 | 0.07 | 82.1 | **<0.001** | 1.64 | 2.16 |
|  |  |  |  |  |  |  |  |  |
|  | Time since the most recent quarantine  (N=55434) | > 4 weeks | 1.27 | 0.08 | 9.47 | **0.002** | 1.09 | 1.47 |
|  |  | 2-4 weeks | 1.47 | 0.13 | 8.06 | **0.005** | 1.13 | 1.91 |
|  |  | < = 2 weeks | 1.39 | 0.05 | 44.3 | **<0.001** | 1.26 | 1.53 |
|  |  |  |  |  |  |  |  |  |
| Probable anxiety | Duration of quarantine (N=53611) | < = 2 weeks | 1.27 | 0.06 | 18.46 | **<0.001** | 1.14 | 1.42 |
|  |  | 2-4 weeks | 1.67 | 0.10 | 27.39 | **<0.001** | 1.38 | 2.03 |
|  |  | > 4 weeks | 2.18 | 0.15 | 28.71 | **<0.001** | 1.64 | 2.89 |
|  | Time since the most recent quarantine  (N=47525) | > 4 weeks | 1.38 | 0.05 | 38.15 | **<0.001** | 1.25 | 1.53 |
|  |  | 2-4 weeks | 1.32 | 0.15 | 3.22 | 0.07 | 0.98 | 1.79 |
|  |  | < = 2 weeks | 1.61 | 0.16 | 9.14 | **0.003** | 1.18 | 2.19 |

Note: Adjusted for age group, gender, education, BMI, current smoking, history of psychiatric disorder, chronic medical conditions, COVID-19 diagnosis, living condition before the latest measurement of mental health indicators, current quarantine and living condition.


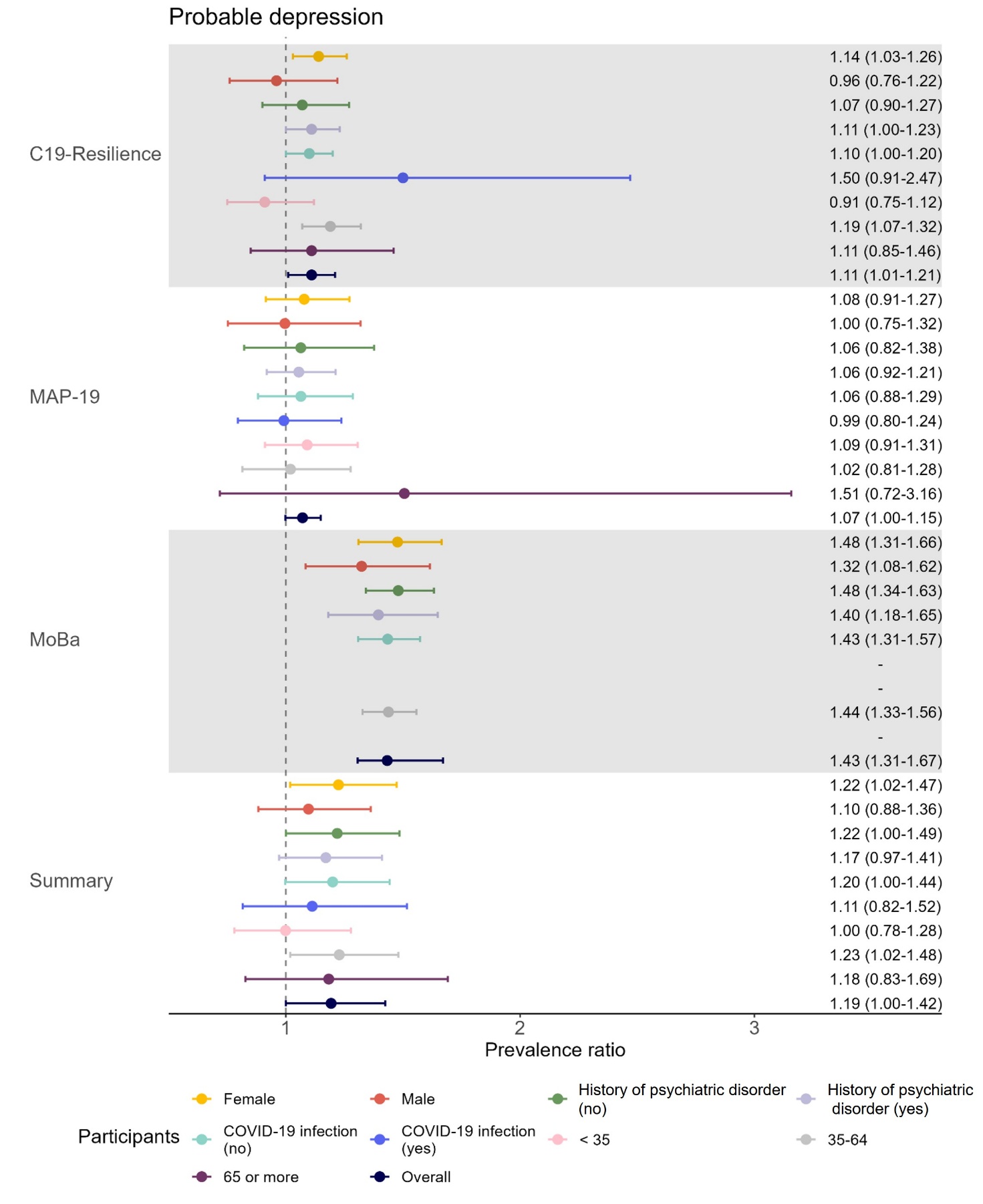
 Fig. S1 Prevalence ratio (PR) and 95% confidence interval (CI) of **probable depression** of participants with ***quarantine experience*** compared with those without quarantine stratified by age group, sex, COVID-19 infection and history of psychiatric disorder overall and in each cohort


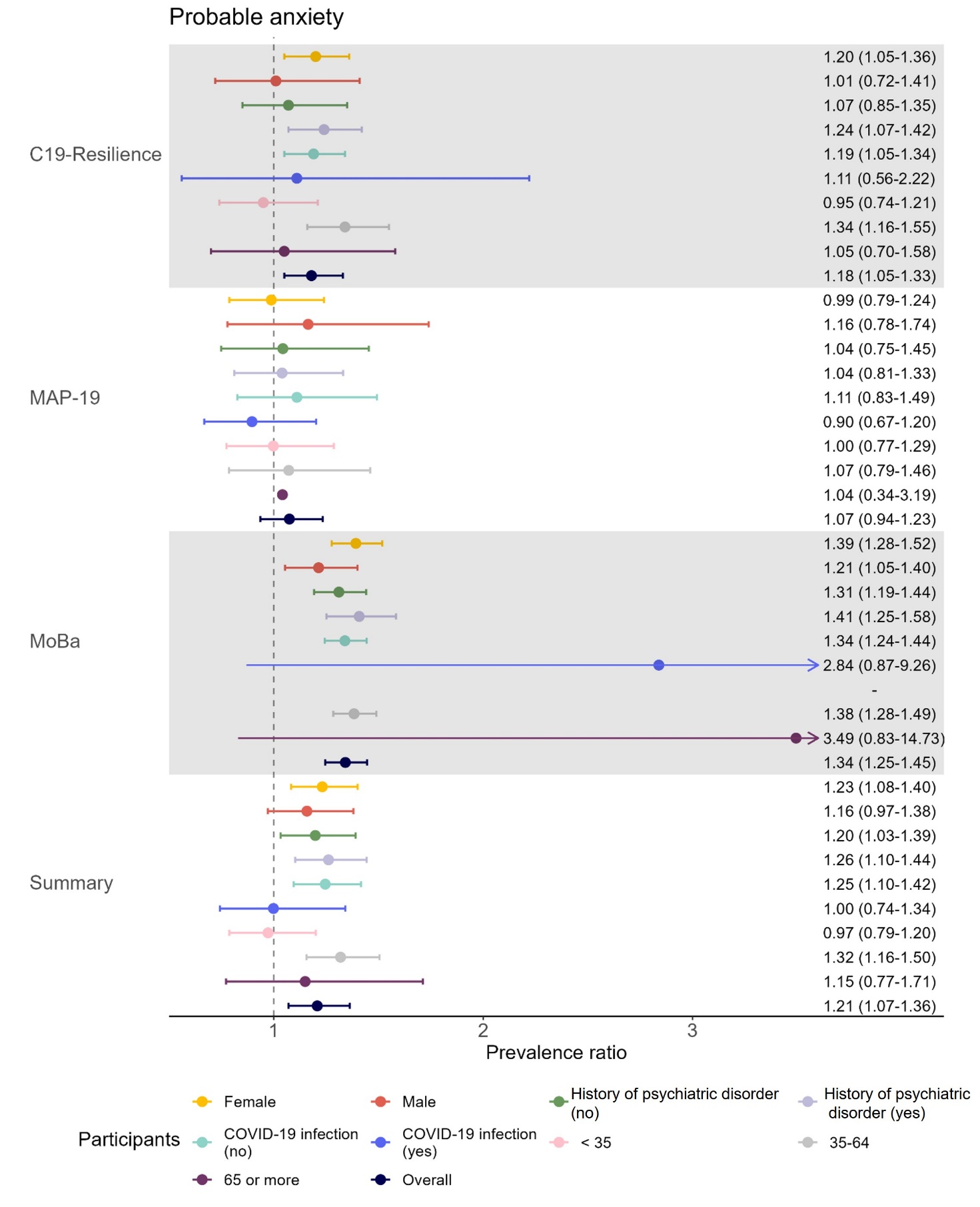
 Fig. S2 Prevalence ratio (PR) and 95% confidence interval (CI) of **probable anxiety** of participants with ***quarantine experience*** compared with those without quarantine stratified by age group, sex, COVID-19 infection and history of psychiatric disorder overall and in each cohort
